# Water-soluble SNS cationic palladium(II) complexes and their Suzuki–Miyaura cross-coupling reactions in aqueous medium

**DOI:** 10.3762/bjoc.14.160

**Published:** 2018-07-23

**Authors:** Alphonse Fiebor, Richard Tia, Banothile C E Makhubela, Henok H Kinfe

**Affiliations:** 1 Department of Chemistry, University of Johannesburg, PO Box 524, Auckland Park 2006, South Africa; 2 Department of Chemistry, Kwame Nkrumah University of Science and Technology, Kumasi, Ghana

**Keywords:** cationic palladium(II) complexes, Pd(II)/Pd(IV) complexes, SNS pincer complexes, Suzuki–Miyaura

## Abstract

Unlike their SCS analogues, SNS pincer complexes are poorly studied for their use in coupling reactions. Accordingly, a series of water soluble cationic Pd(II) SNS pincer complexes have been successfully synthesised and investigated in detail for their catalytic activity in Suzuki–Miyaura coupling reactions. By using only 0.5 mol % loading of the complexes, the coupling of inactivated aryl bromides and activated aryl chlorides with various boronic acids in water was achieved in excellent yields and the catalysts were found to be reusable for three cycles without a significant loss of activity. The investigation of the mechanism of the reaction revealed that a Pd(II) to Pd(IV) route is the more likely pathway which was further supported by computational studies.

## Introduction

**Figure 1:**
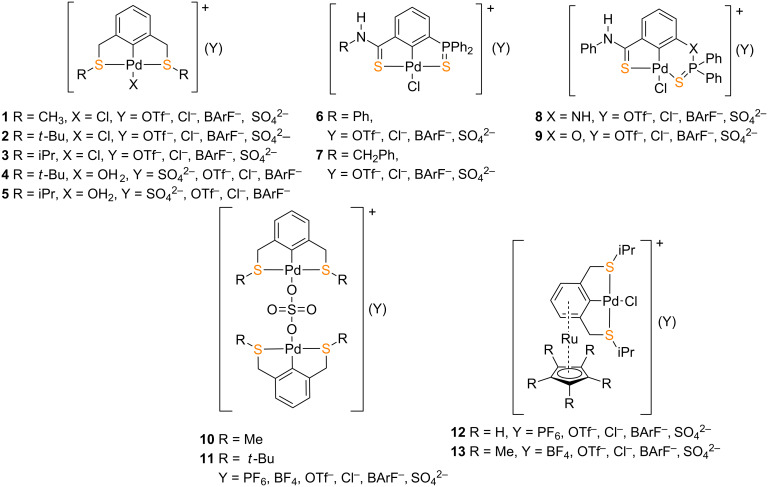
Examples of reported SCS palladium(II) pincer complexes **1–13**.

The Suzuki–Miyaura C–C coupling reaction is a powerful method for the synthesis of ubiquitous biaryls and has been extensively employed in the synthesis of natural products, pharmaceuticals, agrochemicals, and polymers. The reaction usually involves a palladium-catalysed coupling of aryl boronates with aryl halides in organic solvents in the presence of an excess of base [1–3]. With the drive for the development of environmentally friendly and low cost protocols, a number of methodologies for the Suzuki–Miyaura reaction under aqueous conditions or in neat water have been reported [4–6]. This has been achieved via microwave heating [7–15], ultrasonication [16–18], ligand-free methodologies [19–25] and the use of water-soluble palladium pre-catalysts/catalysts [26–30]. The latter is the preferred choice since it allows for the reusability of the catalyst for subsequent reactions after simple phase separation [31]. However, the commonly employed phosphorous and carbene ligand-based palladium(II) complexes are found to be in most cases sensitive to moisture and air limiting the scope of their catalytic application in aqueous reactions [32–33]. This limitation encouraged for the development of organosulfur ligand based palladium(II) complexes by exploiting the strong donor properties of sulfur. Such complexes are found to be resistant to moisture, air and thermal stress/elevated temperatures and have been applied in catalysing Suzuki–Miyaura coupling reactions [32–33]. As it was elegantly reviewed by Singh and co-workers [33], these organosulfur ligands can be classified into pincer type (symmetrical and unsymmetrical), thioethers, thiourea-based ligands, sulfur-substituted NHCs, thiosemicarbazones and sulfated Schiff bases. Of the pincer ligands, there are several examples of SCS-based palladium(II) complexes (**1–13**, Figure 1) which were reported to catalyse the Suzuki–Miyaura coupling reaction but the corresponding easy-to-synthesise SNS pincer complexes are well underrepresented [33].

**Figure 2:**
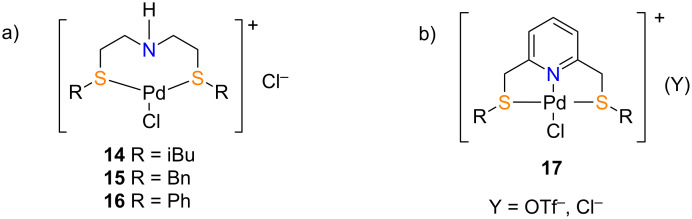
a) Reported SNS palladium(II) pincer complexes **14**–**16** as catalysts for Suzuki–Miyaura cross coupling [34]; b) Proposed SNS palladium(II) pincer complexes **17**.

To the best of our knowledge, the only examples reported in the literature are the water-soluble pincer complexes **14**, **15** and **16** (Figure 2a). While pincer complex **14** provided moderate (38–68%) GC yields over 6 h at 75 °C using 2 mol % catalyst loading with inactivated aryl bromides, pincer complex **15** was found to be incompatible with both activated and inactivated aryl bromides according to the study conducted by Bai and Hor [34]. Similarly, Kumar et al. studied the catalytic activity of pincer complex **16** and reported that the catalyst was compatible with activated aryl bromides to provide reasonable yields over 12 h at 100 °C using 2 mol % catalyst loading; but required stoichiometric amounts of tetra-*n*-butylammonium bromide (TBAB) to effect the reaction of inactivated aryl bromides [35]. A further limitation of these catalysts is their incapability to catalyse the coupling reaction when either activated or inactivated aryl chlorides are employed as coupling partners. Encouraged by the high hydrophilicity and the potential activity of such complexes, we are interested in the synthesis of cationic palladium(II) SNS pincer complexes of the general structure **17** (Figure 2b) having rigid fused cyclic rings imparted by a pyridine backbone; and systematic investigation of their catalytic activity in the aqueous Suzuki–Miyaura coupling reaction. Herein, we report the synthesis of the SNS Pd(II) pincer complexes and their interesting catalytic activities in the Suzuki–Miyaura cross coupling reactions in neat water.

## Results and Discussion

**Scheme 1:**
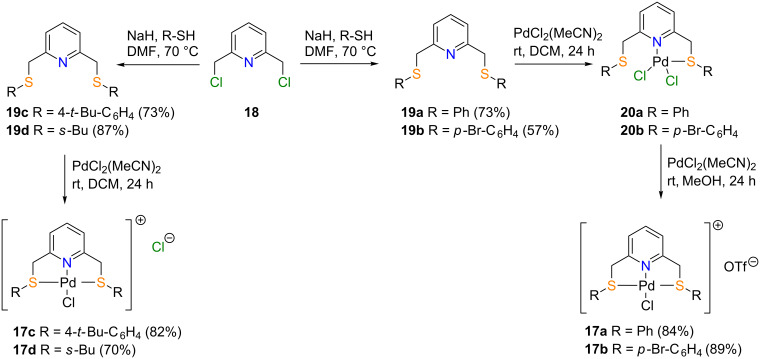
Synthesis of pincer ligands **19a**–**d** and complexes **17a**–**d**.

Our study commenced with the preparation of the SNS pincer ligand **19a** using literature reported protocols [36] as shown in Scheme 1. Treatment of in situ-generated thiophenolate with 2,6-bis(chloromethyl)pyridine (**18**) afforded pincer ligand **19a** in 73% yield. Initial attempts for the synthesis of SNS-Pd(II) complex **17a** (with Cl^−^ counter ion) were unsuccessful since the reaction of the SNS pincer ligand **19a** with PdCl_2_(MeCN)_2_ led to the formation of N,S-Pd(II) **20a** presumably due to the weak basicity of the sulfur atom. Gratifyingly, the problem was solved by treatment of the reaction mixture with the halide abstractor AgOTf to provide the desired SNS-Pd(II) complex **17a** in quantitative yield (Scheme 1).

**Figure 3:**
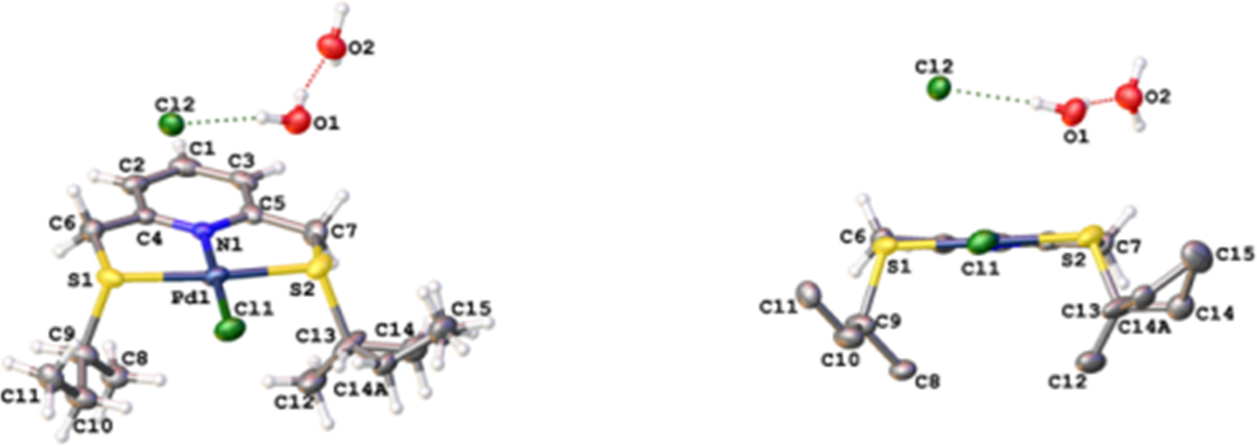
Molecular structure of **17d**. Selected bond distances (Å) and bond angles (°); S(1)–Pd(1)–Cl(1) 93.27(4), S(2)–Pd(1)–Cl(1) 93.34(4), N(1)–Pd(1)–S(1) 86.70(9), N(1)–Pd(1)–S(2) 86.71(9), C(6)–S(1)–C(9) 100.2(2), C(7)–S(2)–C(13) 102.72(2), S(1)–C(6) 1.808(4), S(2)–C(7) 1.816(4), Pd(1)–Cl(1) 2.3044(10).

The structure of the complex was established using NMR spectroscopy and high-resolution mass spectrometry. Among others, the downfield shift of all the protons in the complex, when compared to the corresponding protons of the ligand, by a difference of Δδ_H_ 0.5 to 1.3 suggests the deshielding of the nuclei due to coordination with the palladium. The downfield shift of the protons is in accordance with other previously reported palladium(II) complexes [37]. The symmetry of the complex was supported by the appearance of the two protons of the pyridine moiety as a doublet at δ_H_ 7.76. Furthermore, the formation of the two fused five-membered rings was confirmed by the appearance of the axial and equatorial protons of the bridging methylene groups as two broad singlets at δ_H_ 5.59 and δ_H_ 5.16 each integrating for two protons as opposed to the appearance of the corresponding protons in the ligand as a singlet at δ_H_ 4.29 integrating for four protons. In a similar fashion, complex **17b** was synthesised and characterised successfully while ligands **19c** and **19d** possessing the electron-donating thioether side groups favoured the formation of the corresponding pincer complexes **17c** and **17d** directly without formation of an isolable bidentate intermediate observed during the synthesis of complexes **17a** and **17b**. Furthermore, the successful synthesis of the pincer complexes was confirmed by X-ray crystallography and the representative X-ray crystal structure of **17d** is shown in Figure 3 [38].

Single crystals suitable for X-ray analysis of complexes **17d** were obtained by slow evaporation of a mixture of dichloromethane and diethyl ether at about −4 °C. Crystallographic data and structure refinement parameters for **17d** are summarised in Table 1. Formation of the tridentate pincer complex was confirmed by the crystal structure of **17d**. The structure is accompanied by two lattice water molecules interacting with each other and an uncoordinated chloride ion through hydrogen bonding. The expected square planar geometry of palladium(II) complexes can be observed in the crystal structure of **17d** where the bond angles around the palladium metal centre are 93.27(4)°, 93.34(4)°, 86.70(9)° and 86.71(9)° for S(1)–Pd(1)–Cl(1), S(2)–Pd(1)–Cl(1), N(1)–Pd(1)–S(1) and N(1)–Pd(1)–S(2), respectively. It is observed that the *sec*-butyl groups attached to the sulfur atom are pointing away from the plane of the metal centre such that the thioether angles are 100.2(2)° and 102.72(2)° corresponding to C(6)–S(1)–C(9) and C(7)–S(2)–C(13), respectively. The thioether C–S bond lengths obtained are 1.808(4) Å and 1.816(4) Å for S(1)–C(6) and S(2)–C(7), respectively.

**Table 1:** Crystal data and structure refinement parameters for **17d**.

identification code	Complex **17d**

empirical formula	C_15_H_24_Cl_2_NPdS_2_, 2(H_2_O)
formula weight	124.20
temperature/K	100.01
crystal system	triclinic
space group	P-1
*a*/Å	7.7438(12)
*b/*Å	7.8801(12)
*c*/Å	17.140(3)
α/°	76.788(2)
β/°	89.363(2)
γ/°	82.357(2)
volume/Å^3^	1009.0(3)
*Z*	8
ρ_calc_g/cm^3^	1.635
μ/mm^−1^	1.398
F(000)	508.0
crystal size/mm^3^	0.7 × 0.27 × 0.2
radiation	Mo Kα (λ = 0.71073)
2Θ range for data collection/°	4.884 to 52.232
index ranges	−9 ≤ h ≤ 9, −9 ≤ k ≤ 9, −21 ≤ l ≤ 21
reflections collected	17514
independent reflections	4019 [R_int_ = 0.0199, R_sigma_ = 0.0159]
data/restraints/parameters	4019/0/228
goodness-of-fit on F^2^	1.101
final R indexes [I>=2σ (I)]	R_1_ = 0.0393, wR_2_ = 0.0836
final R indexes [all data]	R_1_ = 0.0410, wR_2_ = 0.0845
largest diff. peak/hole / e Å^−3^	1.83/−1.47

The thioether bond lengths and angles were found to be consistent to the reported data of 1.816 Å and 100.75°, respectively, by Sogukomerogullari et al. [39]*.* Similarly, the Pd–Cl bond length of **17d** (2.3044(10) Å) also corresponds to that of square planar complexes with a chlorine ligand reported in literature [40]. The crystal structure of **17d** also shows a static disorder of a CH_2_ carbon of one of the *sec*-butyl groups (C14). The atom site occupancy factors of the two positions of the CH_2_ carbon atom was refined to 0.571(11) and 0.429(11). This shows that the carbon atom has equal probability of being oriented in the two positions.

With the desired pincer complexes in hand, we then moved on to investigate their potential as catalysts for Suzuki–Miyaura coupling reactions. In our first attempt, the electronically deactivated 4-bromoanisole (**21a**) along with phenylboronic acid (**22a**) as coupling partner and pincer complex **17a** as catalyst were selected, in order to identify the optimum catalyst and reaction conditions (Table 2). Carrying out the reaction with equimolar amounts of the coupling partners in water at 140 °C for 4 hours in the presence of 1 mol % pincer complex **17a** and K_3_PO_4_ (2 equiv) led to the formation of the biaryl **23a** in 92% yield (Table 2, entry 1). The colour of the reaction mixture remained yellow. The use of solvents other than water gave inferior yields (Table 2, entries 2 and 3) which could be attributed to the high hydrophilicity of the pincer complex **17a**. Several bases including pyridine, Et_3_N, KOH, K_2_CO_3_ and Cs_2_CO_3_ were then evaluated. All except Et_3_N provided the expected biaryl **23a** in comparable and excellent yields (Table 2, entries 4–8). The poor performance of Et_3_N as base in the reaction could be due to its low water solubility. KOH as a common laboratory reagent gave a comparable yield to the other bases. Therefore, we opted to use KOH as our preferred base for the reaction. An improved 93% yield was obtained when the reaction was carried out at 120 °C (Table 2, entry 9). However, lowering the reaction temperature to less than 120 °C resulted in comparatively poorer yields (Table 2, entries 10–12). Monitoring of the reaction at different time intervals under otherwise identical conditions showed slightly progressive increase in the yield of the product formed (Table 2, entries 13–15) with a maximum yield of 93% after 4 hours (Table 2, entry 9). However, stirring the reaction mixture beyond 4 hours did not lead to improved yields. Investigation on the loading of the catalyst indicated that 1 mol % was sufficient to drive the reaction to near completion (93% yield, Table 2, entry 9) considering the minor byproduct formed via homocoupling of the phenylboronic acid coupling partner. Reducing the catalyst loading by half yielded a competitive yield of 86% (a 7% decreased yield, Table 2, entry 16). Catalyst loading of less than 0.5 mol % provided inferior yields (Table 2, entries 17 and 18). In agreement with a literature report [27], the addition of TBAB (0.5 equiv) improved the performance of the reaction (Table 2, entries 14 vs 19 and 16 vs 20) but the colour of the reaction mixture turned black.

**Table 2:** Optimization of the Suzuki–Miyaura cross coupling reaction of 4-bromoanisole (**21a**) and phenylboronic acid (**22a**).

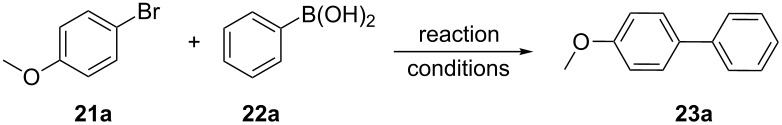								

Entry	Base	Solvent	Cat.	Cat. loading	Temp.	Additive	Time (h)	Yield (%)^a^

**1**	K_3_PO_4_	H_2_O	**17a**	1	140	–	4	92
**2**	K_3_PO_4_	DMF	**17a**	1	140	–	4	67
**3**	K_3_PO_4_	toluene	**17a**	1	140	–	4	0
**4**	Pyr	H_2_O	**17a**	1	140	–	4	90
**5**	Et_3_N	H_2_O	**17a**	1	140	–	4	51
**6**	KOH	H_2_O	**17a**	1	140	–	4	90
**7**	K_2_CO_3_	H_2_O	**17a**	1	140	–	4	95
**8**	Cs_2_CO_3_	H_2_O	**17a**	1	140	–	4	92
**9**	KOH	H_2_O	**17a**	1	120	–	4	93
**10**	KOH	H_2_O	**17a**	1	100	–	4	84
**11**	KOH	H_2_O	**17a**	1	80	–	4	79
**12**	KOH	H_2_O	**17a**	1	20 (rt)	–	4	41
**13**	KOH	H_2_O	**17a**	1	120	–	0.5	77
**14**	KOH	H_2_O	**17a**	1	120	–	1	80
**15**	KOH	H_2_O	**17a**	1	120	–	2	82
**16**	KOH	H_2_O	**17a**	0.5	120	–	4	86
**17**	KOH	H_2_O	**17a**	0.25	120	–	4	46
**18**	KOH	H_2_O	**17a**	0.06	120	–	4	0
**19**	KOH	H_2_O	**17a**	1	120	TBAB	1	91
**20**	KOH	H_2_O	**17a**	0.5	120	TBAB	4	94
**21**	KOH	H_2_O	**17a**	0.5	120	–	2	80
**22**	KOH	H_2_O	**17a**	0.5	120	TBAB	2	85
**23**	KOH	H_2_O	**17c**	0.5	120	–	2	73
**24**	KOH	H_2_O	**17c**	0.5	120	TBAB	2	90
**25**	KOH	H_2_O	**17d**	0.5	120	–	2	72
**26**	**KOH**	**H_2_O**	**17d**	**0.5**	**120**	**TBAB**	**2**	**93**
**27**	KOH	H_2_O	**17b**	0.5	120	–	2	75
**28**	KOH	H_2_O	**17b**	0.5	120	TBAB	2	85
**29**	KOH	H_2_O and Hg	**17a**	0.5	120		2	72
**30**	KOH	H_2_O and Hg	**17a**	0.5	120	TBAB	2	92

^a^GC yield.

Among the set of SNS pincer complexes investigated in this study, pincer complex **17a** was found to be the most active catalyst in the absence of TBAB as an additive (Table 2, entry 21 vs 23, 25 and 27). Such an outcome suggests that the sterically less demanding planar phenyl group renders greater access to the metal centre for the substrates than the bulkier side groups (both with electron-donating and electron-withdrawing substituents) and leads to the enhanced catalytic activity observed for **17a**, which thus implies that the catalytic activity is influenced by steric as opposed to electronic effects. On the contrary, carrying out the reactions under otherwise identical conditions but with the presence of TBAB, the activity of the catalysts was reversed. The electron rich and sterically demanding *tert*-butylphenyl and isobutyl thioether possessing SNS pincer complexes resulted in higher yields (Table 2, entry 24 and 26) than the electronically poor and sterically less demanding phenyl group (Table 2, entry 22) as well as the electronically poor but sterically demanding 4-bromophenyl thioether (Table 1, entry 28) possessing SNS pincer complexes. These results suggest that in the presence of TBAB the mechanism of the reaction changes and the catalytic activity seems to be under the influence of electronic as opposed to steric effects. When the reaction mixture turned black in the presence of TBAB, we were under the impression that the reaction could have proceeded via formation of palladium nanoparticles as it is common with most palladium catalysed coupling reactions. However, mercury drop experiments provided comparable yields (Table 2, entry 29 and 30) under the same reaction conditions ruling out the possibility of a coupling reaction catalysed by palladium nanoparticles [37].

**Scheme 2:**
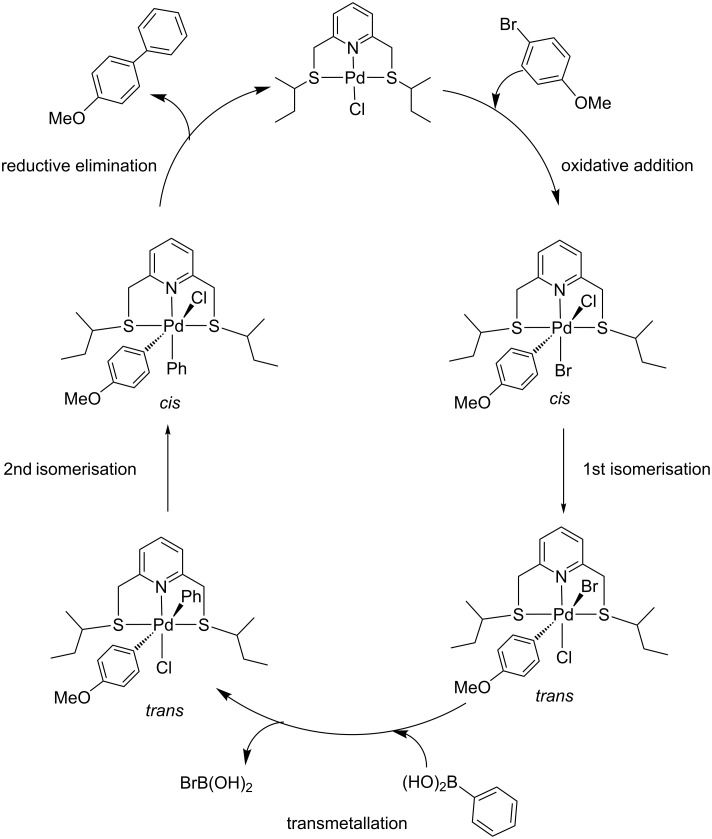
Proposed mechanism of the Suzuki–Miyaura coupling reaction using pincer complex **17d**.

The fact that the reaction mixture remained yellowish in the absence of TBAB and the mercury drop experiments did not lead to an appreciable decrease in yield, the reaction was proposed to proceed via a Pd(II) to Pd(IV) mechanism contrary to the previously reported SNS pincer Pd(II) complexes which proceeded via formation of Pd nanoparticles [35] (Pd(II) to Pd(0) type of mechanism). Literature suggestions on a Pd(II) to Pd(IV) mechanism are known for biscarbene (CNC), alkylphosphine (PCP) and aminophosphine (PCP) pincer complexes, and in each of these cases, catalysis was unaffected by metallic mercury during the cross-coupling reactions [41–43]. In addition, stable Pd(IV) complexes have been widely prepared and characterised, thus for the catalysis of the pincer complexes in the current study, the Pd(II) to Pd(IV) mechanism was proposed as shown in Scheme 2 [44].

In order to verify the viability of the Pd(II) to Pd(IV) mechanism proposed, an exploratory computational study using the semi-empirical PM3 method was performed on the oxidative addition stage of the mechanism to determine if the results obtained theoretically could support the experimental results. The oxidative addition stage was selected because it is also the rate-determining step for the catalytic cycle, and thus it can be used to compare the rate of conversions. The PM3 method has been parameterised for transition metal systems and has been found to give geometries, relative energies and activation energy trends in good agreement with high-level density functional theory (DFT) results at a fraction of the computational cost [45–46]. It is therefore adequate for studies in which the prime purpose is to determine or verify preferred reaction pathways.

**Figure 4:**
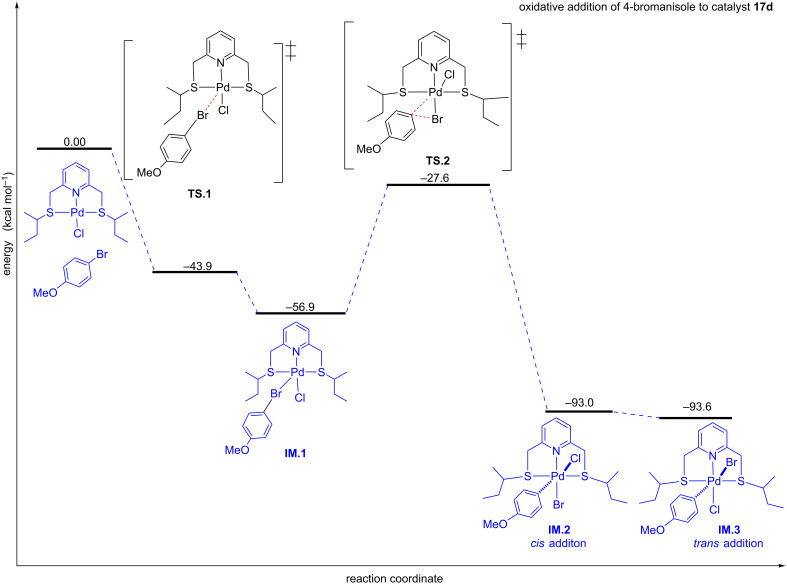
Energy profile for the oxidative addition reaction involving 4-bromoanisole and Pd(II) catalyst precursor **17d**. The change in energy for **17d** was calculated by computing it in kcal/mol as a cation at 298.15 K and 1 atm.

Oxidative addition of 4-bromoanisole to complex **17d** proceeds by the C–Br bond activation to form a weakened Pd···Br bond, of 3.104 Å length in transition state (**TS1**), which ultimately results in a new Pd–Br bond, of 2.605 Å length (**IM1**) (Figure 4). The Pd–Br distance is calculated to be 2.60 Å and is consistent with similar Pd–Br bond lengths found in the literature for single crystal X-ray structures [47–48]. This oxidative addition step involves a low activation energy barrier of −43.9 kcal/mol. This negative energy implies that there is a stable intermediate between the reactants and the transition state (**TS1**). Thus, when the energy of the transition state is lower than the energy of the reactants or intermediates from which the transition state is formed it means there is a stable intermediate between them.

Nonetheless, such a relatively low energy barrier indicates that the reaction proceeds at a fast rate in getting to the intermediate (**IM1**), thus it is kinetically favoured. The reaction then proceeds via transition state **TS2**, leading to the cleavage of the C(sp^2^)–Br bond and the formation of a new Pd–C(sp^2^) bond, to form a *cis* intermediate of energy −93.0 kcal/mol. The new Pd–C(sp^2^) bond distance of 2.00 Å falls within the reported Pd–C(sp^2^) bond distances [47–48]. Since the bromo ligand is larger in atomic radius than the chloro ligands the more stable form of the oxidative addition product is the *trans* form. As such, the *cis* product can isomerise to a more stable *trans* intermediate of energy −93.6 kcal/mol having a Pd–C(sp^2^) bond length of 1.99 Å.

Next, under the optimal conditions with **17d** as a catalyst (since it provided the highest yield in the presence of TBAB, Table 2, entry 26), we examined the substrate scope with different substituents on the aromatic rings of the bromobenzene and phenylboronic acid. The results are summarised in Table 3 and the scope and yields compare favourably well with reported methods. First, the substrate scope of the phenylboronic acid coupling partner was investigated. Under the optimised reaction conditions, the presence of electron-donating and moderately deactivating groups provided slightly better yields and faster reactions than those possessing strongly deactivating substituents (Table 3, entries 2–4 vs 5). Next, the substrate scope of the aryl bromides was studied. Interestingly, the reaction provided excellent yields with both activated and deactivated aryl bromides, though the presence of a strongly electron-withdrawing substituent gave marginally better yields than those possessing electron-donating groups (Table 3, entries 6–11) without much influence on the rate of the reaction. The slightly lower yield of the reaction in the preparation of **23i** could be ascribed to steric encumbrance exerted by the NO_2_-substituent located at the *ortho*-position of the aryl bromide.

**Table 3:** Results from the Suzuki–Miyaura cross-coupling reactions of various aryl bromides and boronic acids using pincer complex **17d** as catalyst.^a^

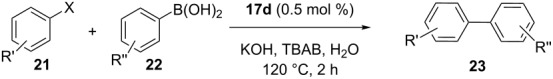						

Entry	ArBr	ArB(OH)_2_	Product	Yield (%)		Time (h)
				GC	Isolated	

**1**	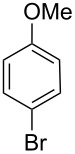 **21a**	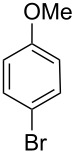 **22a**	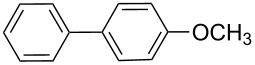 **23a**	93	88	2
**2**	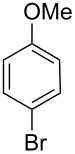 **21a**	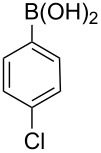 **22b**	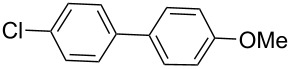 **23b**	96	90	1.5
**3**	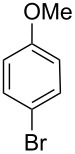 **21a**	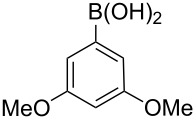 **22c**	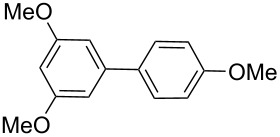 **23c**	94	86	2
**4**	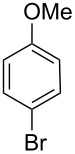 **21a**	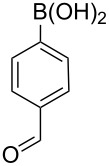 **22d**	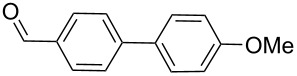 **23d**	92	86	2
**5**	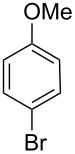 **21a**	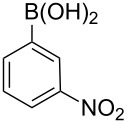 **22e**	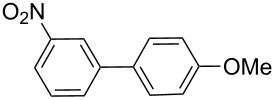 **23e**	87	81	3
**6**	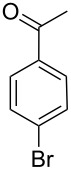 **21b**	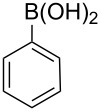 **22a**	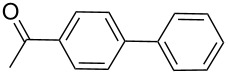 **23f**	98	91	2
**7**	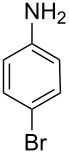 **21c**	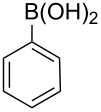 **22a**	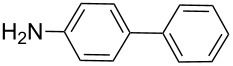 **23g**	96	82	2
**8**	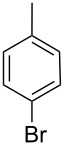 **21d**	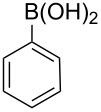 **22a**	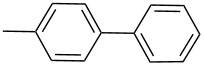 **23h**	96	88	1.5
**9**	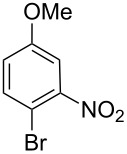 **21e**	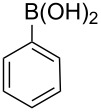 **22a**	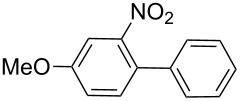 **23i**	94	81	2
**10**	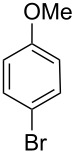 **21a**	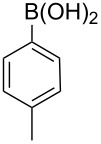 **22f**	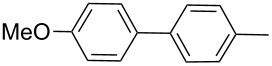 **23j**	96	89	2
**11**	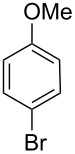 **21a**	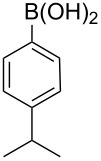 **22g**	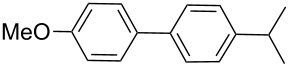 **23k**	95	86	2
**12**	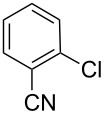 **21f**	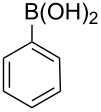 **22a**	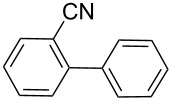 **23l**	83	77	3
**13**	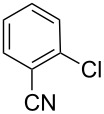 **21f**	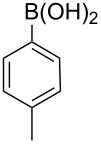 **22f**	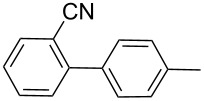 **23m**	89	81	3

^a^Catalyst loading (0.5 mol %), ArX (1.63 mmol), ArB(OH)_2_ (2.61 mmol), H_2_O (2 mL), KOH (3.26 mmol), 120 °C, 2 h, TBAB (0.5 equiv relative to ArX).

Encouraged by these results, the reactivities of aryl chlorides were investigated under the optimised conditions. While activated aryl chloride **21f** reacted with boronic acids possessing either electron-donating or electron-withdrawing groups to provide the corresponding biaryls in reasonable yields, to our dismay the reaction of inactivated aryl chlorides led to the recovery of starting materials (Table 3, entries 12 and 13).

**Scheme 3:**
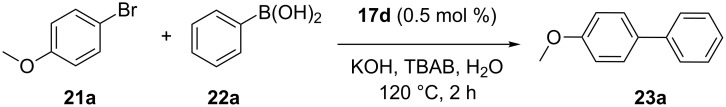
Investigation on the reusability of the catalyst.

**Figure 5:**
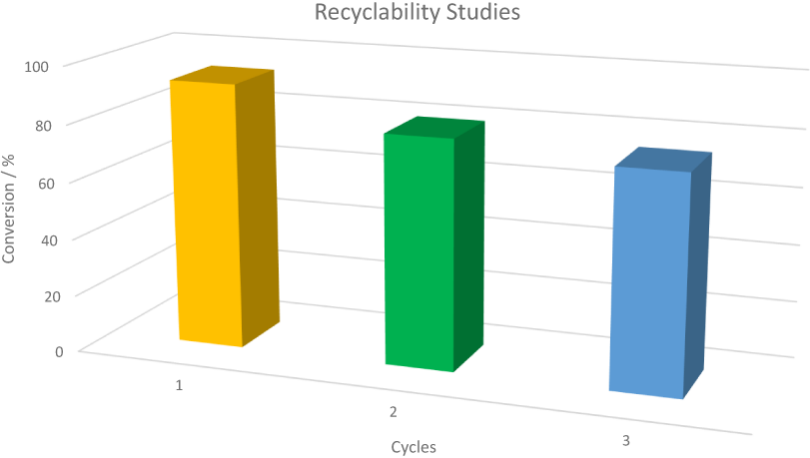
Reusability of pincer complex **17d** as a catalyst for the Suzuki–Miyaura cross coupling reaction.

Finally, the investigation of the reusability of the catalyst was carried out using the model cross-coupling reaction of 4-bromoanisole (**21a**) and phenylboronic acid (**22a**) under the optimised conditions (Scheme 3 and Figure 5). After completion of the reaction, the products were extracted with toluene and to the remaining aqueous layer, which contains the catalyst system, fresh 4-bromoanisole (**21a**) and phenylboronic acid (**22a**) were added for the second and third cycles of the reaction. To our delight, the catalyst could be reused at least three times without significant loss of activity (93%, 80% and 75% for the 1st, 2nd and 3rd run, respectively) considering the fact that some of it could have been extracted along with the product and the results are summarised in Figure 5.

**Scheme 4:**
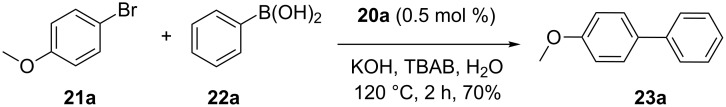
Suzuki–Miyaura coupling reaction catalysed by the SN-bidentate complex **20a**.

Since the bidentate complexes **20a** and **20b** are also potential catalysts in their own right, the catalytic activity of **20a** was investigated in the Suzuki–Miyaura coupling reaction of 4-bromoanisole (**21a**) and phenylboronic acid (**22a**) as a proof of concept. As expected, it catalysed the reaction and provided biaryl **23a** in moderate yield demonstrating the potential of such complexes in coupling reactions (Scheme 4).

## Conclusion

A series of novel cationic Pd(II) complexes have been successfully synthesised using easy to prepare SNS pincer ligands. A detailed investigation into the application of these complexes in the Suzuki–Miyaura coupling reaction was conducted using various aryl bromides and boronic acids as coupling partners in aqueous medium. All the complexes could catalyse the Suzuki–Miyaura coupling reaction to provide the corresponding biaryls in excellent yields with only 0.5 mol % catalyst loading. Furthermore, unlike the previously reported SNS pincer Pd(II) complexes, the catalysts in the current study were compatible with both electron-donating and electron-withdrawing substituents on the aryl bromides and boronic acid substrates as well as activated aryl chlorides. Depending on the presence or absence of the TBAB additive, the reaction may be fine-tuned to either proceed via steric or electronic control. The advantage of using these water-soluble catalysts for the coupling reaction was their reusability for up to three times without significant loss in activity. Moreover, the mechanism of the coupling reaction was probed by a theoretical study that supported the experimental results. Contrary to the previously reported SNS pincer Pd(II) complexes that were proposed to proceed via a Pd(II) to Pd(0) type of mechanism, on the basis of the study described in this article the coupling reaction is proposed to proceed via a Pd(II) to Pd(IV) mechanism. This suggests the effect of the chain length of the linker and the nature of the backbone on the catalytic activity of the SNS pincer Pd(II) complexes. In the future, efforts to fine tune the electronic and structural features of the thiophenyl and pyridinyl groups will be carried out in order to enable the catalysts to activate aryl chlorides, possessing electron-donating substituents, in the coupling reaction.

## Supporting Information

File 1 Experimental part.

File 2 NMR spectra.
