# Supplementary material for: Water-soluble SNS cationic palladium(II) complexes and their Suzuki–Miyaura cross-coupling reactions in aqueous medium
Source: Beilstein J Org Chem. 2018 Jul 23;14:1859–70. doi: 10.3762/bjoc.14.160 (PMC6071702; doi:10.3762/bjoc.14.160)
Supplement: File 1 — Experimental part. [file Beilstein_J_Org_Chem-14-1859-s001.pdf]

Supporting Information

for

Water-soluble SNS cationic palladium(II) complexes and their Suzuki–Miyaura cross-coupling reactions in aqueous medium

Alphonse Fiebor[1], Richard Tia[2], Banothile C. E. Makhubela[1]* and Henok H. Kinfe[1]*

Address: [1]Department of Chemistry, University of Johannesburg, PO Box 524, Auckland Park 2006, South Africa and [2]Department of Chemistry, Kwame Nkrumah University of Science and Technology, Kumasi, Ghana

Email: Banothile Makhubela - bmakhubela@uj.ac.za; Henok Kinfe - hhkinfe@uj.ac.za

* Corresponding author

Experimental part

Experimental

All reactions were carried out under inert conditions unless otherwise stated. 2,6-Bis (chloromethyl)pyridine, the aryl thiols, sodium hydride (60% oil dispersion), the aryl halides, TBAB, and the boronic acid reagents were all purchased from Sigma-Aldrich and were used as supplied. All solvents used were of analytical grade and were dried using MBRAUN SPS-800 solvent drying system as required. $PdCl_2$ was

purchased from Heraeus South Africa. The metal precursor $[PdCl_2(MeCN)_2]$ was synthesized from $PdCl_2$ following reported literature method [1]. NMR spectra were recorded on Bruker 400 MHz NMR spectrometer (^{1}H at 400 MHz and ^{13}C{^{1}H} at 100 MHz). Spectrometer chemical shifts were reported relative to the internal standard tetramethylsilane (δ 0.00 ppm). Elemental analysis was performed on a Thermos Scientific FLASH 2000 CHNS-O Analyzer. Mass spectrometry was performed using Waters Synapt G2 mass spectrometer with both ESI positive and Cone Voltage 15 V. For the optimization conditions, the reactions were monitored by GC. GC analyses for all the biaryls were carried using a Scion 456-GC, with 30 mm × 0.25 mm cyanopropylphenylmethylpolysiloxane phase column set to an initial temperature of 80 °C and then increased to 300 °C at 10 °C/min. All reactions were monitored by thin-layer chromatography (TLC) using silica gel coated aluminium plates (0.2 mm). Developed plates were visualized with UV light or under iodine staining. Silica gel column chromatography was performed using silica gel 60 (70–230 mesh). FTIR spectra were recorded using a PerkinElmer FT-IR Spectrum BX-ATR.Melting points were determined using a Gallenkamp digital melting point apparatus.

General procedure for the synthesis of ligands 19a–d

To a solution of aryl/alkyl thiol (1.71 mmol) in DMF (3 mL), sodium hydride (2.05 mmol) was added. The reaction mixture was stirred for 30 min (or until evolution of gas ceased) under the atmosphere of nitrogen. 2,6-Bis(chloromethyl)pyridine (0.57 mmol) was then added to the yellowish reaction mixture and the resulting mixture was stirred at 70 °C. After TLC analysis indicated completion of reaction (≈10 h), water (30 mL) was added to quench the reaction. The aqueous solution was then extracted with ethyl acetate (3 × 20 mL). The combined organic layers were then

dried over $MgSO_4$, concentrated in vacuo and the crude product was purified by column chromatography using a mixture of ethyl acetate/hexane (1:9) as eluent to provide ligands **19a–d**:

2,6-Bis((phenylthio)methyl)pyridine (19a)

73% yield; pale yellow oil; FT-IR (*vmax*/cm^{-1}): 737 (C-S), 1736 (C=N), 3057 (=C-H); ^{1}H NMR (400 MHz, DMSO-d_6) δ: 7.66 (t, *J* = 7.6 Hz, 1H, Ar), 7.35 (d, *J* = 8.0 Hz, 4H, 2 × AA'-part of AA'XX'- system of Ar), 7.17 (t, *J* = 7.6 Hz, 2H, Ar), 7.27-7.24 (m, 6H, Ar), 4.29 (s, 4H, 2 × CH_2); $^{13}C\{^{1}H\}$ NMR (100 MHz, $CDCl_3$) δ: 158.2, 157.3, 157.2 (pyridine), 137.1, 135.8, 135.7, 133.9, 132.4, 132.3, 129.4, 129.3, 128.8, 127.7, 126.2, 121.8, 121.2, 120.4 (Ar), 60.9, 40..2 (CH_2); HR-ESI-MS $C_{19}H_{18}NS_2$ calcd m/z = 324.0881 [M+H], found m/z = 324.0884 $[M+H]^+$. Elemental analysis: Anal. Calcd. CHNS for: C, 70.55%; H, 5.30%; N, 4.33%; S, 19.83%; Found C, 70.63%; H, 5.06%; N, 3.74%; S, 19.53%.

2,6-Bis(((4-(*tert*-butyl)phenyl)thio)methyl)pyridine (19c)

73% yield; off-white oil; FT-IR (*vmax*/cm^{-1}): 828 (C-S), 1739 (C=N), 2970 (=C-H); ^{1}H NMR (400 MHz, DMSO-d_6) δ: 7.57 (t, *J*= 7.6 Hz, 1H, Ar), 7.29-7.22 (m, 10H, Ar), 4.24 (s, 4H, 2 × CH_2), 1.19 (s, 18 H, 6 × CH_3); $^{13}C\{^{1}H\}$ NMR (100 MHz, $CdCl_3$) δ: 157.3, 149.4, 136.9 (pyridine), 132.2, 129.6, 125.7, 121.1 (Ar), 34.2, 31.1 (CH_3), 40.5 (CH_2); HR-ESI-MS $C_{27}H_{33}NS_2$ calcd. m/z = 436.2133 $[M+H]^+$, found m/z = 436.2141 $[M+H]^+$. Elemental analysis: Anal. Calcd. CHNS C, 74.43%; H, 7.63%; N, 3.21%; S, 14.72%; Found C, 73.67%; H, 7.58%; N, 2.17%; S, 15.37%.

2,6-Bis((*sec*-butylthio)methyl)pyridine (19d)

87% yield; pale yellow oil; FT-IR (*vmax/*cm^{-1}): 747 (C-S), 1739 (C=N), 2970 (=C-H); ^1H NMR (400 MHz, $CDCl_3$) δ: 7.60 (t, *J*= 7.6 Hz, 1H, Ar), 7.26 (d, *J* = 7.6 Hz, 2H, AA'-part of AA'XX'-system of Ar), 3.82 (s, 4H, 2 × CH_2), 2.71-2.62 (m, 2H, 2 × -SCH-), 1.65-1.52 (m, 4H, 2 × -$CHCH_2CH_3$), 1.27-1.21 (m, 6H, 2 × CH_3), 0.92-0.89 (m, 6H, 2 × CH_3); $^{13}C\{^1H\}$ NMR (100 MHz, $CdCl_3$) δ: 158.4, 137.4, 136.9, 127.2, 120.7 (pyridine), 36.6, 20.4, 11.0 (CH_3), 41.4, 41.2, 29.4, 29.3 (CH_2); HR-ESI-MS $C_{15}H_{25}NS_2$ calcd. m/z = 284.1507 $[M+H]^+$, found m/z = 284.1513 $[M+H]^+$. Elemental analysis: Anal. Calcd. for CHNS: C, 63.55%; H, 8.89%; N, 4.94%; S, 22.62; Found C, 62.91%; H, 8.75%; N, 4.82%; S, 21.98%.

2,6-Bis(((4-bromophenyl)thio)methyl)pyridine (19b)

57% yield; yellow oil; FT-IR (*vmax/*cm^{-1}): 803 (C-S), 1736 (C=N), 3059 (=C-H); ^1H NMR (400 MHz, DMSO-d_6) δ: 7.67 (t, *J*= 7.6 Hz, 1H, Ar), 7.42 (d, *J* = 8.4 Hz, 4H, 2 × AA'-part of AA'XX'- system of Ar), 7.29-7.26 (m, 6H, Ar), 4.29 (s, 4H, 2 × CH_2); $^{13}C\{^1H\}$ NMR (100 MHz, DMSO-d_6) δ: 156.8, 131.8, 130.1 (pyridine), 137.8, 135.7, 121.7, 118.8 (Ar), 38.3 (CH_2); HR-ESI-MS $C_{19}H_{15}Br_2NS_2$ calcd. m/z = 481.9247 $[M+3H]^+$, found m/z = 481.9069 $[M+3H]^+$. Elemental analysis: Anal. Calcd. for CHNS: C, 47.42%; H, 3.14%; N, 2.91%; S, 13.33%; Found C, 47.74%; H, 3.17%; N, 2.63%; S, 13.93%.

General procedure for the synthesis of pincer complexes 17a and 17b

To a schlenk tube containing a solution of [$PdCl_2$ $(MeCN)_2$] (0.31 mmol) in DCM (5 mL), ligands **19a** or **19b** (0.31 mmol) was added. The reaction mixture was stirred for 24 h at room temperature under inert atmosphere. The solvent was then removed by

vacuum to afford an orange solid bidentate product. A solution of the bidentate product in methanol (5 mL) was treated with AgOTf (0.31 mmol) and stirred for additional 24 h at room temperature. Filtration of the AgCl formed with a micro filter followed by concentration of the filtrate in vacuo afforded the corresponding pincer complexes:

Chloro-2,6-bis((phenylthio)methyl)pyridinepalladium(II) triflate (17a)

84% yield; dark yellow solid; m.p 100-105 °C; FT-IR (*vmax*/cm^{-1}): 634 (C-S), 1602 (C=N), 3087 (=C-H); ^{1}H NMR (400 MHz, DMSO-d_6) δ: 8.21 (t, *J*= 8.0 Hz, 1H, Ar), 7.89 (d, *J* = 6.8 Hz, 4H, 2 × AA'-part of AA'XX' of Ar), 7.76 (d, *J* = 7.6 Hz, 2H, AA'-part of AA'XX'-system of Ar), 7.59-7.51 (m, 6H, Ar), 5.59 (bs, 2H, AA'-part of AA'XX'-system of CH_2), 5.16 (bs, 2H, AA'-part of AA'XX'- system of CH_2) ; $^{13}C\{^1H\}$ NMR (100 MHz, DMSO-d_6) δ: 162.9, 131.1, 130.6 (pyridine), 141.4, 131.1, 129.3, 123.1, 122.4 (Ar); HR-ESI-MS $C_{19}H_{19}NClPdS_2CF_3SO^-{}_3$ calcd. m/z = 465.9682 $[M+2H]^+$, found m/z = 465.9514 $[M+H]^+$. Elemental analysis; Anal. Calcd. for CHNS: C, 39.10%; H, 2.79%; N, 2.28%; S, 15.66%; Found C, 39.04%; H, 2.74%; N, 2.25%; S, 15.61%.

Chloro-2,6-bis(((4-bromophenyl)thio)methyl)pyridinepalladium(II) triflate (17b)

89% yield; dark yellow powder; m.p 170-172 °C; FT-IR (*vmax*/cm^{-1}): 633 (C-S), 1701 (C=N), 3085 (=C-H); ^{1}H NMR (400 MHz, DMSO-d_6) δ: 8.22 (t, *J*= 8.0 Hz, 1H, Ar), 7.83 (d, *J* = 8.4 Hz, 4H, 2 × AA'-part of AA'XX'-system of Ar), 7.29-7.26 (m, 6H, Ar), 5.57 (bs, 2H, AA'-part of AA'XX'- system of CH_2), 5.20 (bs, 2H, AA'-part of AA'XX'-system of CH_2); $^{13}C\{^1H\}$ NMR (100 MHz, DMSO-d_6) δ: 162.5, 133.2, 132.7 (pyridine), 141.2, 131.8, 130.9, 130.5, 130.4, 129.2, 128.6, 124.9, 123.3, 123.2 (Ar) ; HR-ESI-MS $C_{19}H_{11}NBr_2ClPdS_2CF_3SO^-{}_3$ calcd.m/z = 615.7423 $[M-4H]^+$, found m/z =

615.1411 [M-4H]$^+$. Elemental analysis; Anal. Calcd. for CHNS C, 31.11%; H, 1.96%; N, 1.81%; S, 12.46%; Found C, 31.08%; H, 1.93%; N, 1.77%; S, 12.40%.

General procedure for the synthesis of complexes 17c and 17d

To a schlenk tube containing a solution of [$PdCl_2$ (MeCN) $_2$](0.31 mmol) in DCM (5 mL), ligands **19c** or **19d** (0.31 mmol) was added. The reaction mixture was stirred for 24 h at room temperature under inert atmosphere. The solvent was then removed by vacuum to afford the corresponding pincer complexes

Chloro-2,6-bis(((4-(*tert*-butyl)phenyl)thio)methyl)pyridinepalladium(II) chloride (17c)

82% yield; orange solid; m.p 137-140 ^oC; FT-IR (*vmax*/cm^{-1}): 550 (C-S), 1739 (C=N), 2970 (=C-H); ^1H NMR (400 MHz, DMSO-d_6) δ: 8.23 (t, *J*= 8.0 Hz, 1H, Ar), 7.78-7.76 (m, 6H, Ar), 7.56 (d, *J*= 8.4 Hz, 4H, 2 × AA'-part of AA'XX'- system of Ar), 5.39 (s, 4 H, 2 × CH_{2-}), 1.27 (s, 18 H, 6 × CH_3); $^{13}C\{^1H\}$ NMR (100 MHz, DMSO-d_6) δ: 162.9, 154.3, 130.9 (pyridine), 141.3, 129.1, 127.5, 126.1, 125.9, 123.3 (Ar), 34.9, 30.9 (CH_3), 50.1 (CH_2); HR-ESI-MS $C_{27}H_{35}NClPdS_2$ calcd.m/z = 578.0934 [M+2H]$^+$, found m/z = 578.0778 [M+2H]$^+$. Elemental analysis; Anal. Calcd. for CHNS C, 52.90%; H, 5.43%, N, 2.28%, S, 10.46%; Found C, 52.86%; H, 5.40%; N, 2.23%, S, 10.41%.

Chloro-2,6-bis((*sec*-butylthio)methyl)pyridinepalladium(II) chloride (17d):

70% yield; orange solid; m.p 98-100 ^oC; FT-IR (*vmax*/cm^{-1}): 599 (C-S), 1739 (C=N), 3437 (=C-H); ^1H NMR (400 MHz, $CDCl_3$) δ: 8.04-7.96 (m, 3 H, Ar), 5.10 (s, 4H, 2 × CH_2), 3.48-3.43 (m, 2H, 2 × -SC*H*CH_3), 2.10-1.98 (m, 4H, 2 × -CHC$H_2$$CH_3$), 1.91-1.86 (m, 6H, 2 × CH_3), 1.08-0.88 (m, 6H, 2 × CH_3); $^{13}C\{^1H\}$ NMR (100 MHz, DMSO-d_6) δ: 163.9, 140.5, 122.5 (pyridine), 50.3, 28.1, 11.3 (CH_3), 42.9 (CH_2); HR-ESI-MS

$C_{15}H_{27}NClPdS_2$ calcd.m/z = 426.0308 $[M+2H]^+$, found m/z = 426.0144 $[M+2H]^+$. Elemental analysis; Anal. Calcd. for CHNS: C, 39.10%; H, 5.47%; N, 3.04%; S, 13.92%; Found C, 39.06%; H, 5.44%; N, 2.99%; S, 13.87%.

X-ray data collection, structure resolution and refinement of 17d

Crystallization of **17d** was performed by dissolving 5 mg of **17d** in a vial containing about 1 mL dichloromethane followed by the addition of about 4 drops of diethyl ether. The vial was closed with a lid pierced with a needle holes and kept in a refrigerator to allow slow evaporation. After three days, orange irregular crystals were obtained. A suitable single crystal was selected and mounted on a Bruker APEX-II CCD diffractometer. The crystal was kept at 100.01 K during data collection. Data reduction was performed using SAINT+ [2], and the intensities were corrected for absorption using SADABS [2]. All non-hydrogen atoms were refined with anisotropic displacement coefficients. All hydrogen atoms were placed in geometrically idealised positions and constrained to ride on their parent atoms. Using the Olex2 software, the structure was solved with the ShelXT [3] structure solution program using Intrinsic Phasing and refined with the ShelXL [4] refinement package using Least Squares minimisation.

General Suzuki–Miyaura cross-coupling procedure

A 50-mL Schlenk tube was charged with aryl halide (1.63 mmol), aryl boronic acid (2.61 mmol), KOH (3.26 mmol), complex 17d (0.5 mol %), TBAB (0.5 mol % relative to the aryl halide) and water (2 mL). The sealed mixture was stirred at 120 °C until TLC analysis confirmed completion of the reaction (for GC analysis the products were extracted with toluene). The aqueous solution was then extracted with ethyl acetate (3 × 10 mL). The combined organic phases were dried over $MgSO_4$, filtered and evaporated in vacuo. The residue product was purified by column

chromatography on silica gel using a mixture of hexane and ethyl acetate as eluent to yield biaryls **23a–m**:

Representative gas chromatogram after Suzuki–Miyaura cross-coupling reaction

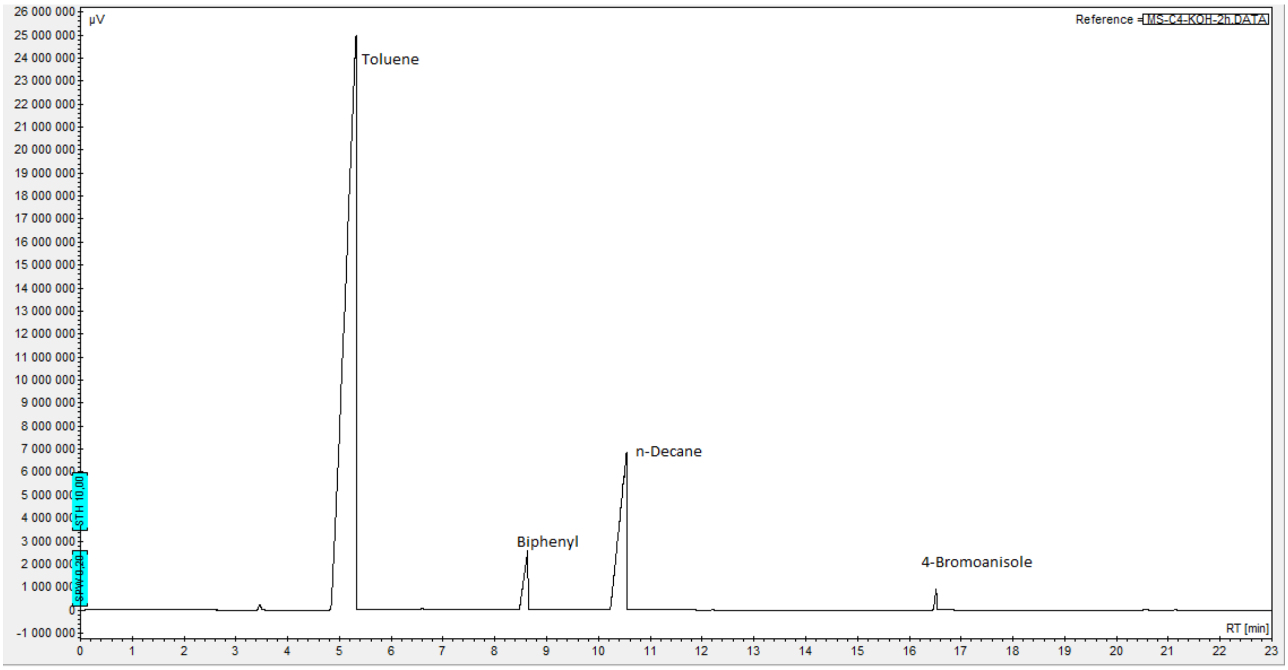

4-Methoxyl-1,1-biphenyl (23a)

88% yield: white solid; m.p 86-88 ºC; ^{1}H NMR (400 MHz, $CDCl_3$) δ: 7.58-7.53 (m, 4 H, Ar), 7.45 (d, *J* = 7.6 Hz, 2 H, AA'-part of AA'XX'- system of Ar), 7.00 (d, *J* = 8.8 Hz, 2H, AA'-part of AA'XX'- system of Ar), 7.43 (t, *J* = 7.6 Hz, 1H, Ar), 3.85 (s, 3 H, OCH3); $^{13}C\{^1H\}$ NMR (100 MHz, $CdCl_3$) δ: 159.1, 158.6, 140.8, 133.7, 132.2, 128.7, 128.1, 126.7, 115.7, 114.2 (Ar), 55.3 (OCH_3). The spectroscopic data were in agreement with the literature reported data [5, 6].

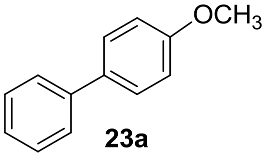

23a

4-chloro-4'-methoxy-1,1'-biphenyl (23b)

90% yield; off-white solid; m.p 109-111 ^{o}C; ^{1}H NMR (400 MHz, $CDCl_3$) δ: 7.49-7.46 (m, 4H, Ar), 7.39 (d, *J* = 8.4 Hz, 2H, AA'-part of AA'XX'- system of Ar), 6.99 (d, *J* = 8.8 Hz, 2H, AA'-part of AA'XX'-system of Ar), 3.85 (s, 3H, OCH_3); $^{13}C\{^1H\}$ NMR (100 MHz, $CdCl_3$) δ: 160.1, 146.9, 134.6, 131.9, 130.2, 128.4, 126.9, 114.4 (Ar), 55.3 (OCH_3). The spectroscopic data were in agreement with the literature reported data [5].

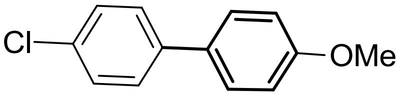

23b

3,4',5-Trimethoxy-1,1'-biphenyl (23c)

86% Yield; off-white solid; m.p 68-70 ^{o}C; ^{1}H NMR (400 MHz, $CDCl_3$) δ: 7.57 (d, *J* = 8.4 Hz, 2H, Ar), 7.00 (d, *J* = 8.8 Hz, 2H, AA'-part of AA'XX'-system of Ar), 6.76 (d, *J* = 2.4 Hz, 2H, AA'-part of AA'XX'- system of Ar), 6.49 (s, 1H, Ar), 3.86 (s, 9H,

OCH_3); $^{13}C\{^1H\}$ NMR (100 MHz, $CdCl_3$) δ: 160.9, 159.3, 142.9, 133.5, 128.1, 114.0, 104.9, 98.6 (Ar), 55.2 (OCH_3). The spectroscopic data were in agreement with the literature reported data [7].

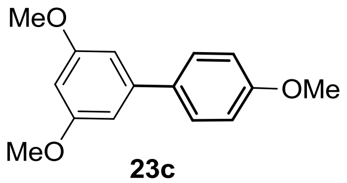

23c

4'-Methoxy-[1,1'-biphenyl]-4-carbaldehyde (23d)

86% yield; white solid; m.p 122-123 ^oC; ^1H NMR (400 MHz, $CDCl_3$) δ: 10.0 (s, 1H, CHO), 7.90 (d, *J* = 8.2 Hz, 2H, AA'-part of AA'XX'- system of Ar), 7.69 (d, *J* = 8.2 Hz, 2H, AA'-part of AA'XX'- system of Ar), 7.57 (d, *J* = 7.0 Hz, 2H, AA'-part of AA'XX'- system of Ar), 6.99 (d, *J* = 7.0 Hz, 2H, AA'-part of AA'XX'-system of Ar), 3.83 (s, 3H, OCH_3); $^{13}C\{^1H\}$ NMR (100 MHz, $CdCl_3$) δ: 159.3, 158.6, 139.2, 132.6, 132.4, 132.2, 128.8, 127.9, 115.7, 114.8, 112.7 (Ar), 55.2 (OCH_3). The spectroscopic data were in agreement with the literature reported data [7].

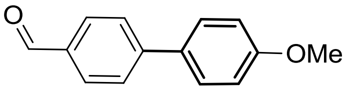

23d

4'-Methoxy-3-nitro-1,1'-biphenyl (23e)

81% yield; pale yellow oil; ^1H NMR (400 MHz, $CDCl_3$) δ: 8.38 (s, 1H, Ar), 8.13 (d, *J* = 12.0 Hz, 1H, Ar), 7.86 (d, *J* = 12.0 Hz, 1H, Ar), 7.57-7.53 (m, 3H, Ar), 7.01 (d, *J* = 8.0 Hz, 2H, AA'-part of AA'XX'- system of Ar), 3.85 (s, 3H, OCH_3); $^{13}C\{^1H\}$ NMR (100 MHz, $CdCl_3$) δ: 171.1, 160.1, 148.7, 142.4, 132.5, 131.0, 129.6, 128.2, 121.3, 114.6 (Ar), 55.4 (OCH_3). The spectroscopic data were in agreement with the literature reported data [8].

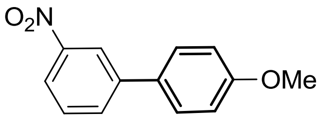

23e

1-([1,1'-Biphenyl]-4-yl)ethanone (23f)

91% yield; white solid; m.p 127-129 ^{o}C; ^{1}H NMR (400 MHz, $CDCl_3$) δ: 8.03 (d, *J* = 8.0 Hz, 2H, AA'-part of AA'XX'- system of Ar), 7.68 (d, *J* = 8.0 Hz, 2H, AA'-part of AA'XX'- system of Ar), 7.62 (d, *J* = 8.0 Hz, 1H, Ar), 7.48 (t, *J* = 7.6 Hz, 2H, Ar), 7.39 (t, *J* = 7.6 Hz, 2H, Ar), 2.63 (s, 3H, CH_3); $^{13}C\{^{1}H\}$ NMR (100 MHz, $CdCl_3$) δ: 145.3, 139.5, 135.5, 128.7, 128.6, 127.9, 126.9, 126.9 (Ar), 26.3 (C=O). The spectroscopic data were in agreement with the literature reported data [5].

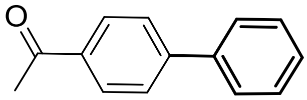

23f

[1,1'-Biphenyl]-4-amine (23g)

82% yield; dark yellow oil; ^{1}H NMR (400 MHz, $CDCl_3$) δ: 7.31 (t, *J* = 8.0 Hz, 1 H, Ar), 6.79 (t, *J* = 8.8 Hz, 2H, Ar), 7.55-7.40 (m, 4H, Ar), 7.57 (d, *J* = 8.4 Hz, 2H, AA'-part of AA'XX'- system of Ar), 3.74 (s, 2H, NH_2); $^{13}C\{^{1}H\}$ NMR (100 MHz, $CdCl_3$) δ: 145.8, 141.1, 131.6, 128.6, 127.9, 126.4, 126.2, 115.4 (Ar). The spectroscopic data were in agreement with the literature reported data [6].

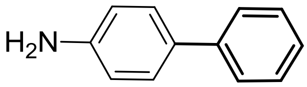

23g

4-Methyl-1,1'-biphenyl (23h)

88% Yield; off-white oil; ^{1}H NMR (400 MHz, $CDCl_3$) δ: 7.64 (d, *J* = 7.6 Hz, 2H, Ar), 7.54 (d, *J* = 8.0 Hz, 2H, AA'-part of AA'XX'- system of Ar), 7.46 (d, *J* = 7.6 Hz, 2H, AA'-part of AA'XX'- system of Ar), 7.41 (t, *J* = 6.6 Hz, 1 H, Ar), 7.29 (d, *J* = 7.6 Hz, 2H, AA'-part of AA'XX'- system of Ar), 2.43 (s, 3H, CH_3); ^{13}C{^{1}H} NMR (100 MHz, $CdCl_3$) δ: 141.2, 138.4, 137.0, 129.5, 128.7, 126.9, 126.9, (Ar), 21.1 (CH_3). The spectroscopic data were in agreement with the literature reported data [5].

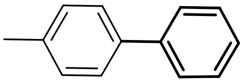

23h

4-Methoxy-2-nitro-1,1'-biphenyl (23i)

81% Yield; pale yellow oil; ^{1}H NMR (400 MHz, $CDCl_3$) δ: 7.29 (s, 1H, Ar), 7.08 (d, *J* = 7.0 Hz, 1H, Ar), 6.91 (d, *J* = 6.0 Hz, 1H, Ar), 7.26-7.19 (m, 5H, Ar), 3.81 (s, 3H, OCH_3); ^{13}C{^{1}H} NMR (100 MHz, $CdCl_3$) δ: 159.2, 137.3, 135.5, 132.8, 128.6, 128.0, 127.8, 119.9, 118.7, 110.7, 108.9, 104.6 (Ar), 56.0 (OCH_3). The spectroscopic data were in agreement with the literature reported data [7].

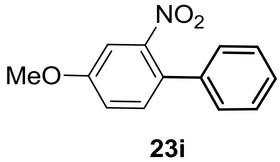

23i

4-Mehoxy-4-methyl-biphenyl (23j)

89% Yield; white solid; m.p 102-104 ºC; ^{1}H NMR (400 MHz, $CDCl_3$) δ: 7.40 (d, *J* = 6.8 Hz, 2H, AA'-part of AA'XX'- system of Ar),7.34 (d, *J* = 8.0 Hz, 2H, AA'-part of AA'XX'- system of Ar), 7.11 (d, *J* = 8.0 Hz, 2H, AA'-part of AA'XX'- system of Ar), 6.85 (d, *J* = 8.8 Hz, 2H, AA'-part of AA'XX'- system of Ar), 3.70 (s, 3H, OCH_3), 2.26

(s, 3H, CH_3) ppm.; $^{13}C\{^1H\}$ NMR (100 MHz, $CdCl_3$) δ: 158.9, 137.9, 136.3, 133.6, 129.4, 127.9, 129.7, 126.7, 126.5, 114.1 (Ar), 55.2 (OCH_3), 21.0 (CH_3) . The spectroscopic data were in agreement with the literature reported data [9].

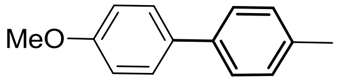

23j

4-Isopropyl-4'-methoxy-1,1'-biphenyl (23k)

86% Yield; off-white solid; m.p 99-101 ^oC; ^1H NMR (400 MHz, $CDCl_3$) δ: 7.59-7.54 (m, 4H, Ar), 7.35 (d, *J* = 8.0 Hz, 2H, AA'-part of AA'XX'- system of Ar), 7.03 (d, *J* = 8.8 Hz, 2H, AA'-part of AA'XX'- system of Ar), 3.02-2.97 (m, 1H, -C**H**CH_3CH_3), 3.88 (s, 3H, OCH_3), 1.36 (s, 6H, CH_3) ppm.; $^{13}C\{^1H\}$ NMR (100 MHz, $CdCl_3$) δ: 158.9, 147.3, 138.4, 133.8, 127.9, 127.7, 126.8, 126.6, 114.1 (Ar), 55.3 (OCH_3), 33.7, 24.0 (CH_3). The spectroscopic data were in agreement with the literature reported data [9].

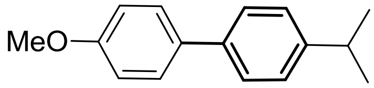

23k

[1,1'-Biphenyl]-2-carbonitrile (23l)

77% Yield; off-white oil; ^1H NMR (400 MHz, $CDCl_3$) δ: 7.67 (d, *J* = 8.0 Hz, 2H, AA'-part of AA'XX'- system of Ar), 7.65-7.47 (m, 5H, Ar), 7.34-7.33 (m, 2H, Ar); $^{13}C\{^1H\}$ NMR (100 MHz, $CdCl_3$) δ: 136.9, 133.9, 133.8, 133.7, 132.8, 130.0, 128.7, 128.7, 128.0, 127.5, 127.1, 113.4 (Ar), 115.9 (CN), The spectroscopic data were in agreement with the literature reported data [10].

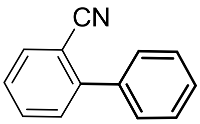

23l

4'-Methyl-[1,1'-biphenyl]-2-carbonitrile (23m)

81% Yield; off-white oil; ^{1}H NMR (400 MHz, $CDCl_3$) δ: 7.63 (d, *J* = 6.8 Hz, 1H, Ar), 7.29 (d, *J* = 8.0 Hz, 2H, AA'-part of AA'XX'- system of Ar), 7.72 (d, *J* = 8.0 Hz, 1H, Ar), 7.51-7.43 (m, 3H, Ar), 7.60 (d, *J* = 6.8 Hz, 1H, Ar), 2.40 (s, 3H, -CH_3); $^{13}C\{^1H\}$ NMR (100 MHz, $CDCl_3$) δ: 145.5, 138.7, 133.9, 133.8, 132.7, 132.7, 130.0, 129.9, 129.4, 128.6, 127.2, 127.1 (Ar), 111.2 (CN), 21.2 (CH_3). The spectroscopic data were in agreement with the literature reported data [10].

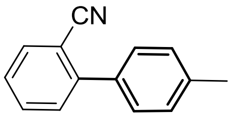

23m

Catalyst reusability procedure

The 50 mL Schlenk tube was charged with pre-catalyst **17d** (0.5 mol %), phenylboronic acid (2.61 mmol), aryl halide (1.63 mmol), TBAB (0.5 mol % relative to the aryl halide), KOH (3.26 mmol) and water (2 mL). The sealed mixture was stirred at 120 °C for 2 h. The reaction mixture was then allowed to cool down to room temperature and 2 mL of toluene was added and this was stirred for additional 5 minutes. The organic layer was separated and analysed using gas chromatography. The aqueous layer which contained the pre-catalyst, was transferred to a clean Schlenk tube for the next reaction cycle.

Mercury drop test

The 50-mL Schlenk tube was charged with pre-catalyst **17d** (0.5 mol %), phenylboronic acid (2.61 mmol), aryl halide (1.63 mmol), TBAB (0.5 mol % relative to the aryl halide), KOH (3.26 mmol), metallic mercury (10 mg) and water (2 mL). The sealed mixture was stirred at 120 °C for 2 h. The reaction mixture was then allowed to cool down to room temperature and 2 mL of toluene was added and this was stirred for additional 5 minutes. The organic layer was separated and analysed using gas chromatography.

Reference:

1. Ocansey, E.;Darkwa, J. Makhubela, B. C. E. *RSC Adv.,* **2018**,*8,* 13826-13834.

2. APEX2 (including SAINT and SADABS), Bruker AXS Inc., Madison, WI, 2012.

3. Sheldrick, G. M. *ActaCryst*. **2015**, *A71*, 3–8.

4. Sheldrick, G. M. *ActaCrystallogr. C Struct. Chem*.,**2015**, *71,* 3–8.

5. Balsane, K. E.; Shendage, S. S.; Nagarkar, J. M. *J. Chem. Sci.,***2015**, *127*, 425-431.

6. Alacid, E.; Nájera, C. *Org. Lett.*, **2008**, *10*, 5011–5014.

7. Mao, S. L.; Yu, G. A.; Zhao, C.; Han, Z. J.; Yuan, J.; Zhu, X.; Yang, Q.; Liu, S. H. *Org. Biomol. Chem.*, **2012**,*10*, 9410-9417.

8. Shi, X.; Cai, C. *New J. Chem.*, **2018**,*42*, 2364-2367.

9. Wang, Z. Y.; Chen, G. Q.; Shao, L. X. *J. Org. Chem.,***2012**, *77*, 6608-6614.

10. Antonacci, G.; Ahlburg, A.; Fristrup, P.; Norrby, P. O.; Madsen, R. *Eur. J. Org. Chem.***2017**, 4758-4764.
