# Supplementary material for: Water-soluble SNS cationic palladium(II) complexes and their Suzuki–Miyaura cross-coupling reactions in aqueous medium
Source: Beilstein J Org Chem. 2018 Jul 23;14:1859–70. doi: 10.3762/bjoc.14.160 (PMC6071702; doi:10.3762/bjoc.14.160)

Supporting Information

for

Water-soluble SNS cationic palladium(II) complexes and their Suzuki–Miyaura cross-coupling reactions in aqueous medium

Alphonse Fiebor[1], Richard Tia[2], Banothile C. E. Makhubela[1]* and Henok H. Kinfe[1]*

Address: [1]Department of Chemistry, University of Johannesburg, PO Box 524, Auckland Park 2006, South Africa and [2]Department of Chemistry, Kwame Nkrumah University of Science and Technology, Kumasi, Ghana

Email: Banothile Makhubela - bmakhubela@uj.ac.za; Henok Kinfe - hhkinfe@uj.ac.za

* Corresponding author

NMR spectra

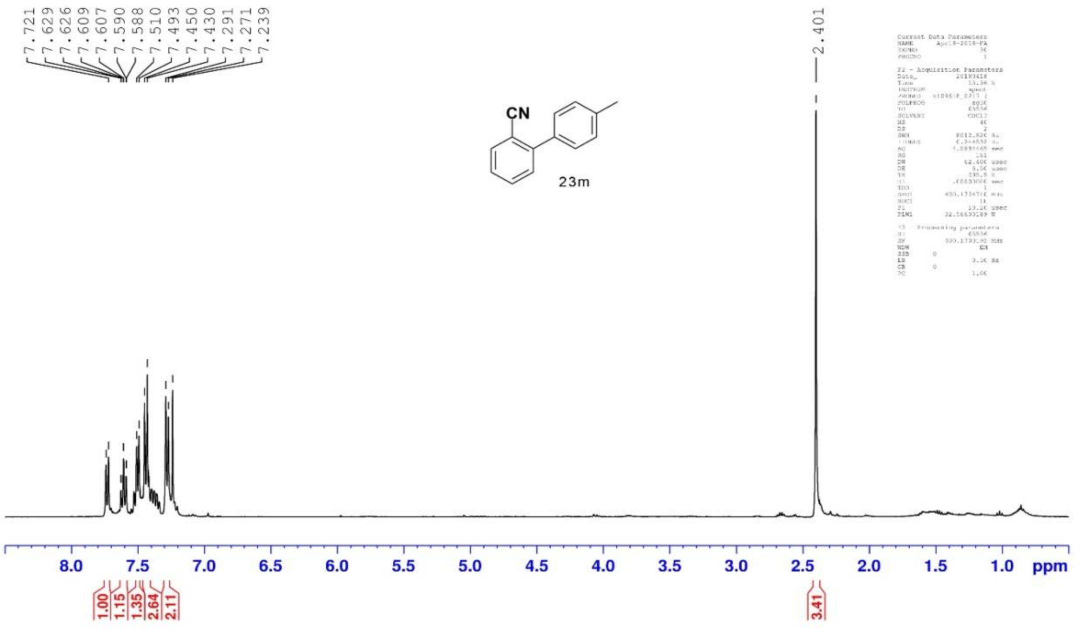
7.721
7.629
7.626
7.609
7.607
7.590
7.588
7.510
7.493
7.450
7.430
7.291
7.271
7.239
2.401
CN
23m
8.0 7.5 7.0 6.5 6.0 5.5 5.0 4.5 4.0 3.5 3.0 2.5 2.0 1.5 1.0 ppm
1.00
1.15
1.35
2.64
2.11
3.41

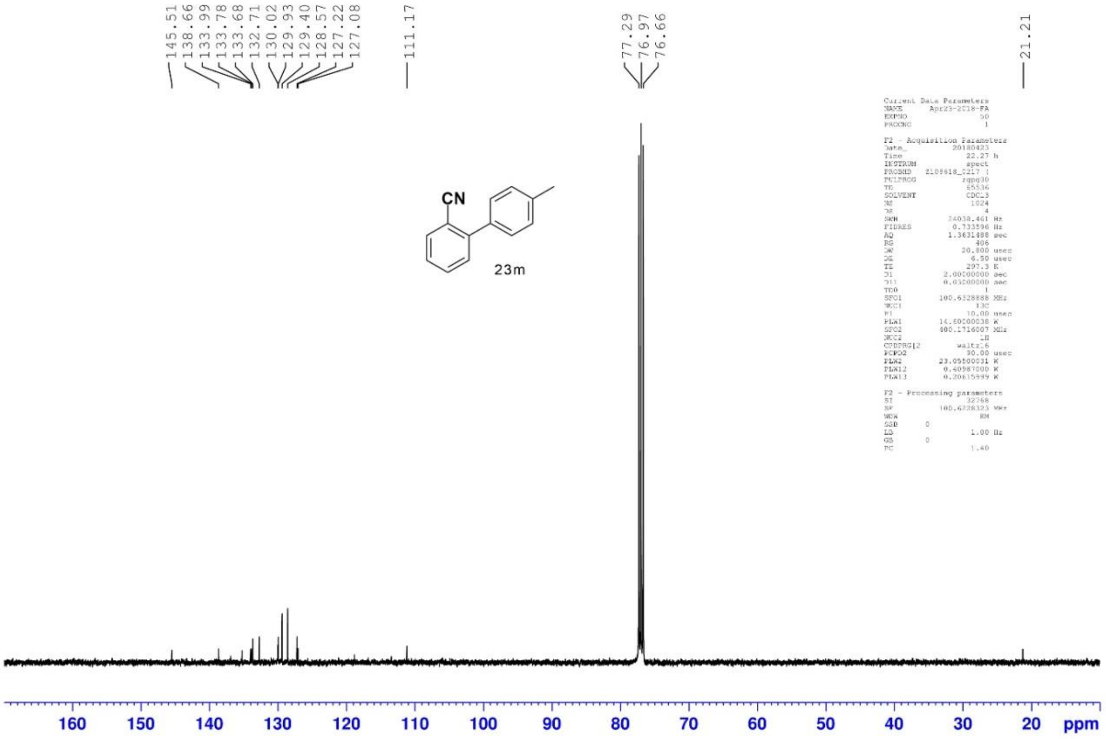
145.51
138.66
133.99
133.78
133.68
132.71
130.02
129.93
129.40
128.57
127.22
127.08
111.17
77.29
76.97
76.66
21.21
CN
23m
160 150 140 130 120 110 100 90 80 70 60 50 40 30 20 ppm

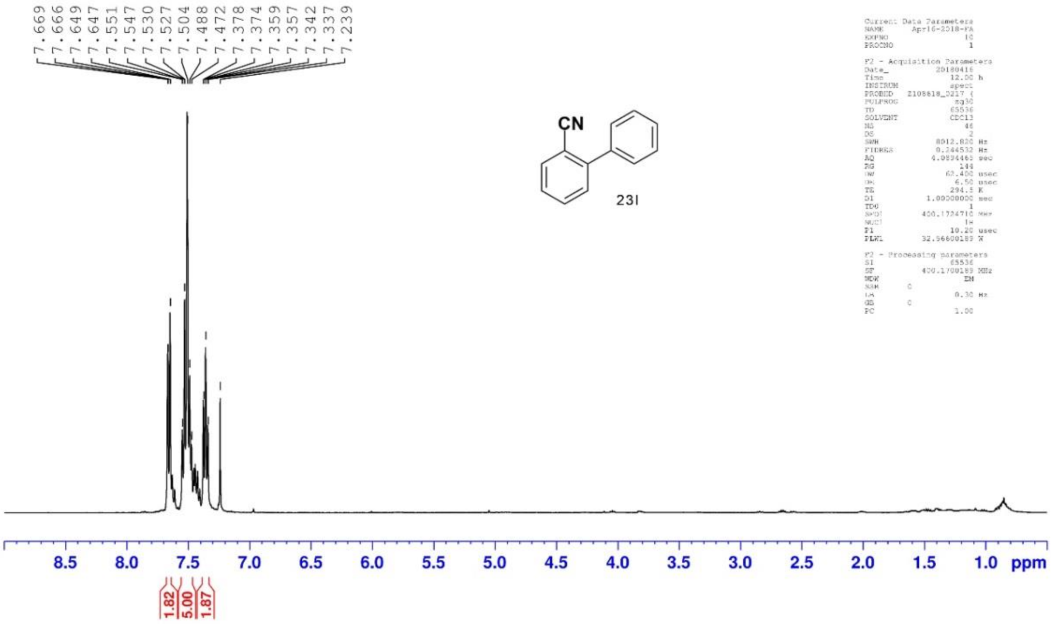
7.669
7.666
7.649
7.647
7.551
7.547
7.530
7.527
7.504
7.488
7.472
7.378
7.374
7.359
7.357
7.342
7.337
7.239
CN
23l
8.5 8.0 7.5 7.0 6.5 6.0 5.5 5.0 4.5 4.0 3.5 3.0 2.5 2.0 1.5 1.0 ppm
1.82
5.00
1.87

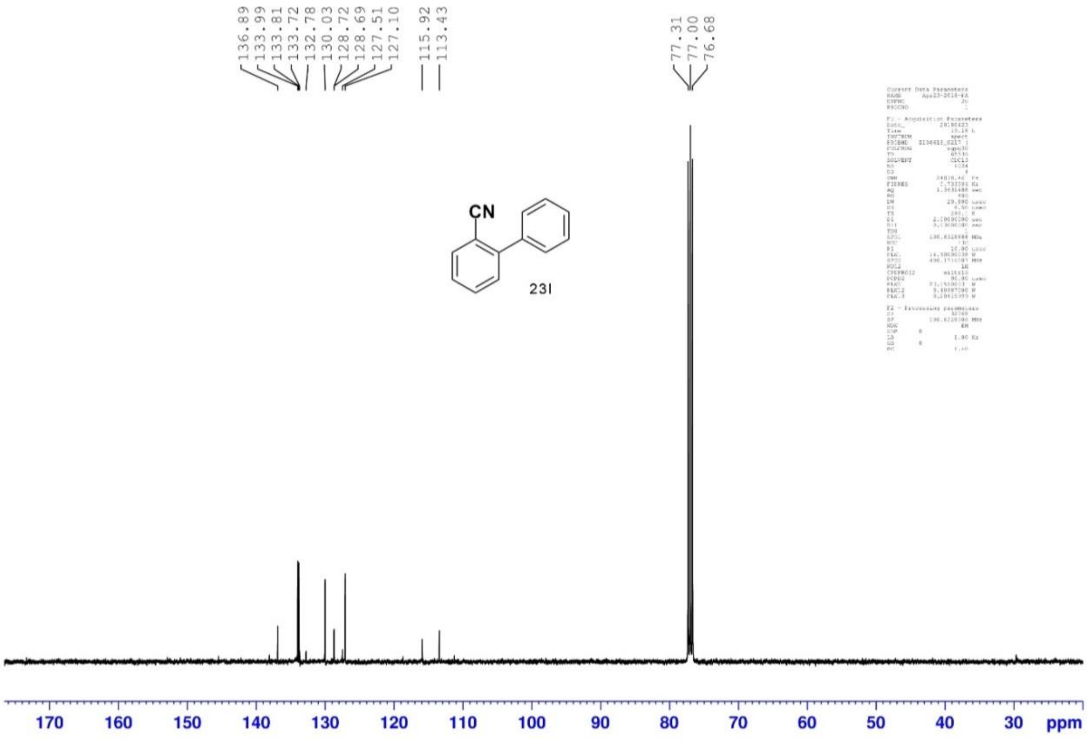
136.89
133.99
133.81
133.72
132.78
130.03
128.72
128.69
127.51
127.10
115.92
113.43
77.31
77.00
76.68
CN
23l
170 160 150 140 130 120 110 100 90 80 70 60 50 40 30 ppm

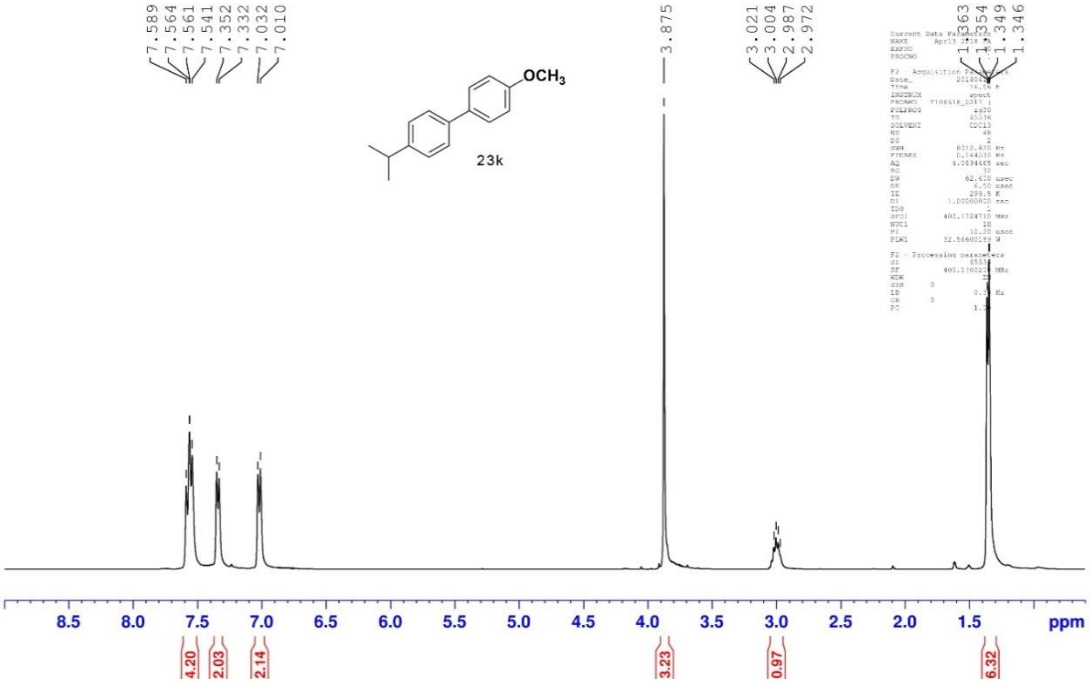
7.589
7.564
7.561
7.541
7.352
7.332
7.032
7.010
3.875
3.021
3.004
2.987
2.972
1.363
1.354
1.349
1.346
OCH3
23k
8.5 8.0 7.5 7.0 6.5 6.0 5.5 5.0 4.5 4.0 3.5 3.0 2.5 2.0 1.5 ppm
4.20
2.03
2.14
3.23
0.97
6.32

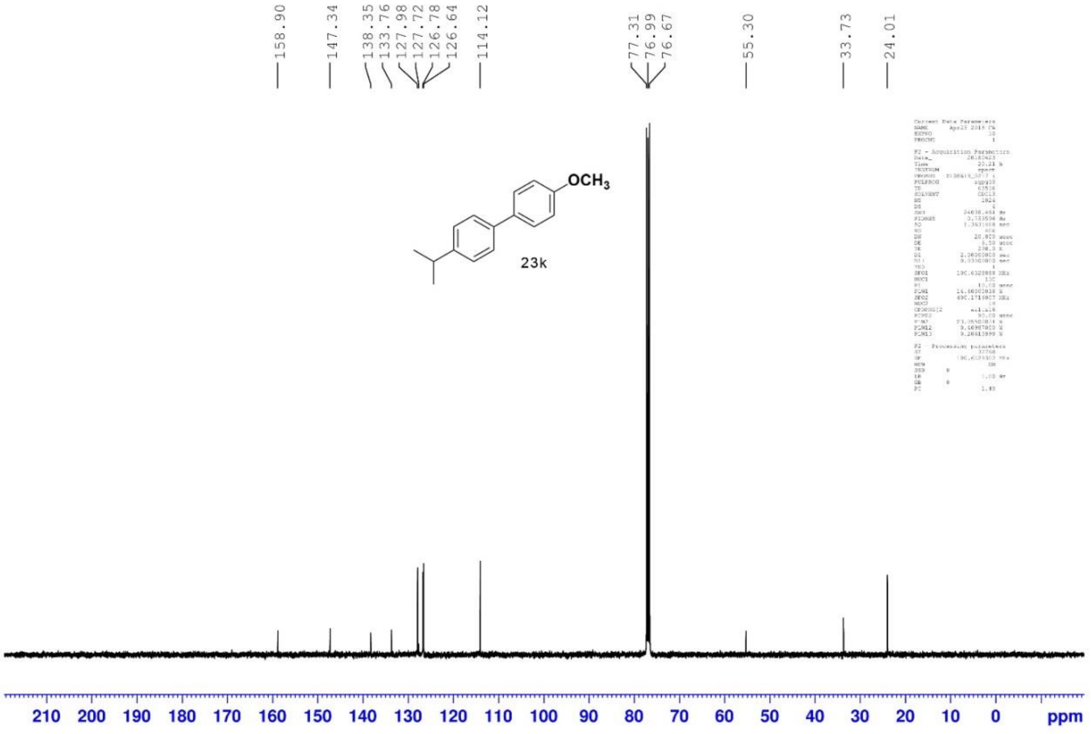
158.90
147.34
138.35
133.76
127.98
127.72
126.78
126.64
114.12
77.31
76.99
76.67
55.30
33.73
24.01
OCH3
23k
210 200 190 180 170 160 150 140 130 120 110 100 90 80 70 60 50 40 30 20 10 0 ppm

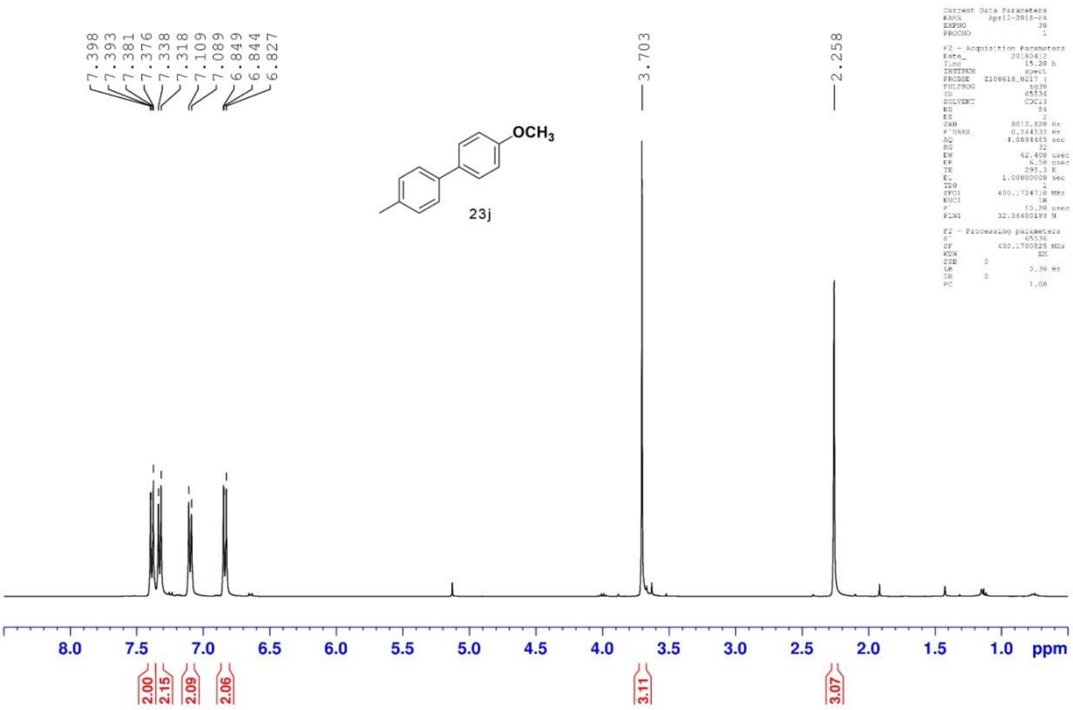
7.398
7.393
7.381
7.376
7.338
7.318
7.109
7.089
6.849
6.844
6.827
3.703
2.258
OCH3
23j
8.0 7.5 7.0 6.5 6.0 5.5 5.0 4.5 4.0 3.5 3.0 2.5 2.0 1.5 1.0 ppm
2.00
2.15
2.09
2.06
3.11
3.07

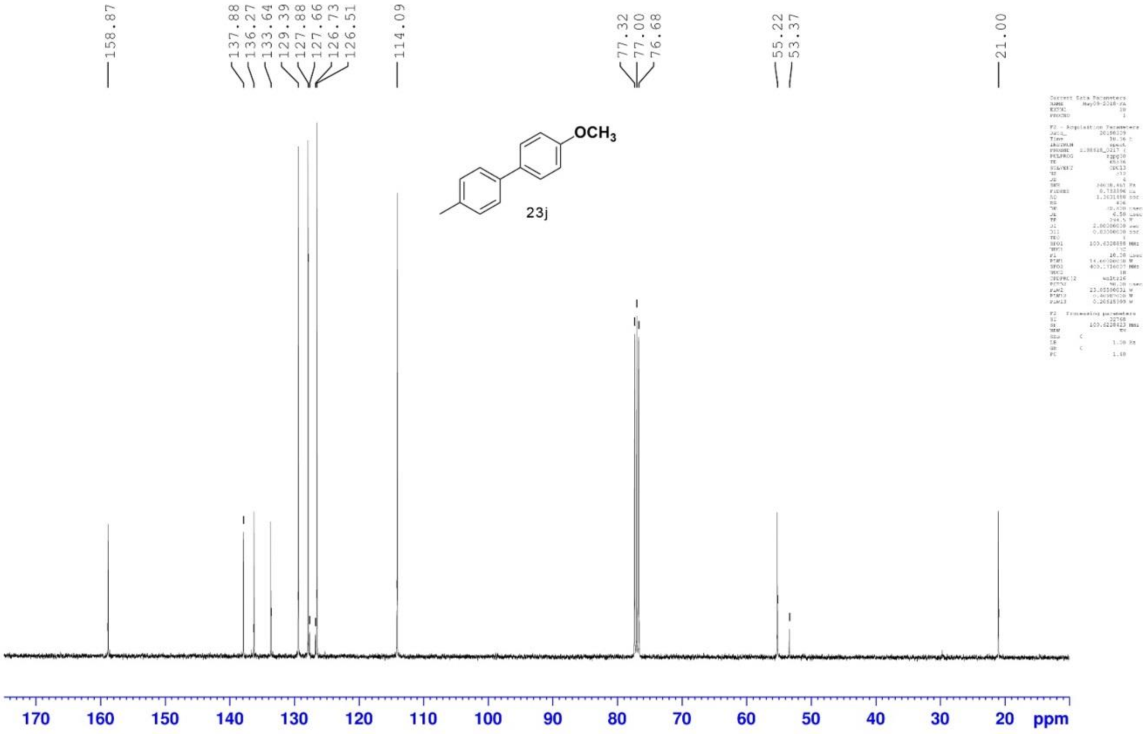
158.87
137.88
136.27
133.64
129.39
127.88
127.66
126.73
126.51
114.09
77.32
77.00
76.68
55.22
53.37
21.00
OCH3
23j
170 160 150 140 130 120 110 100 90 80 70 60 50 40 30 20 ppm

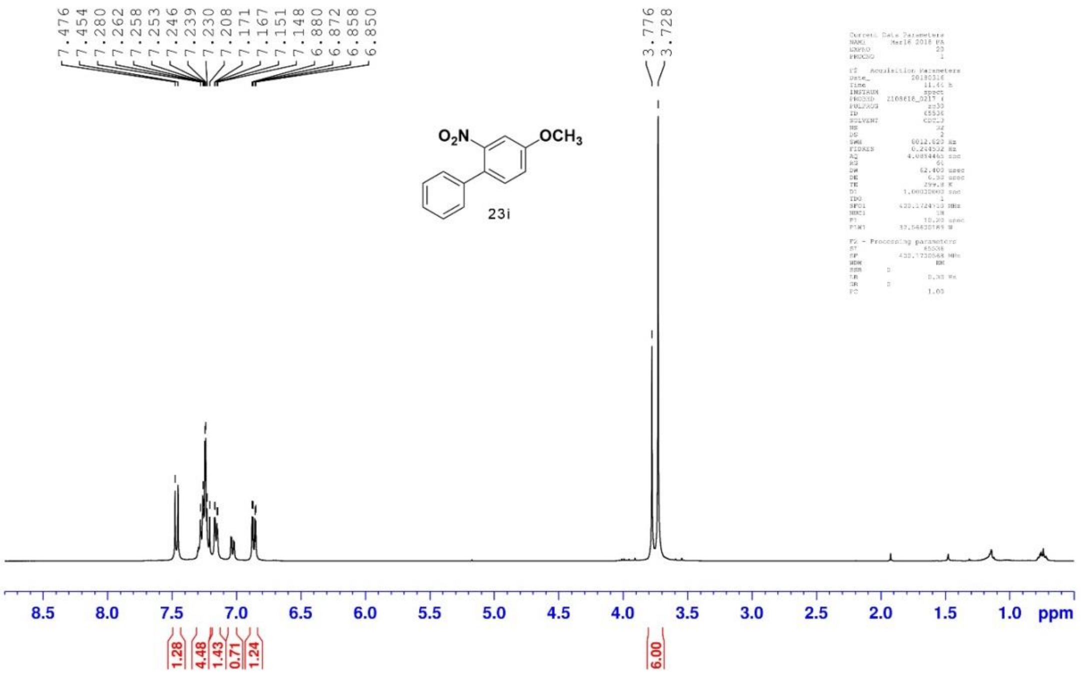
7.476
7.454
7.280
7.262
7.258
7.253
7.246
7.239
7.230
7.208
7.171
7.167
7.151
7.148
6.880
6.872
6.858
6.850
3.776
3.728
O2N
OCH3
23i
8.5 8.0 7.5 7.0 6.5 6.0 5.5 5.0 4.5 4.0 3.5 3.0 2.5 2.0 1.5 1.0 ppm
1.28
4.48
1.43
0.71
1.24
6.00

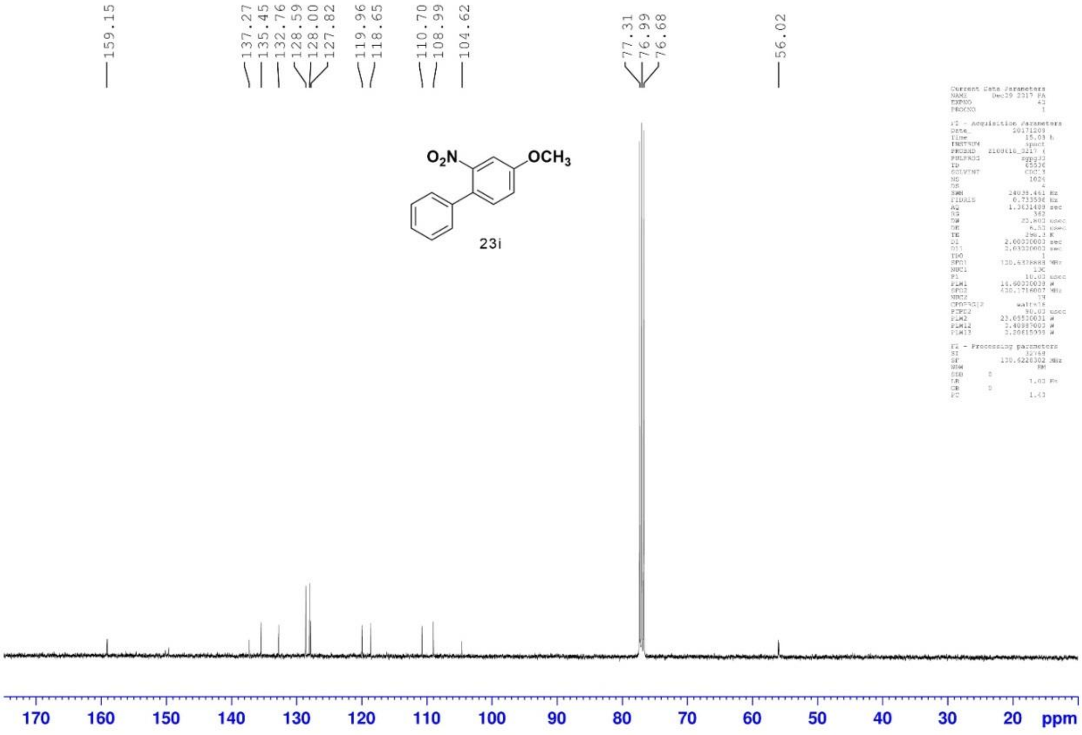
159.15
137.27
135.45
132.76
128.59
128.00
127.82
119.96
118.65
110.70
108.99
104.62
77.31
76.99
76.68
56.02
O2N
OCH3
23i
170 160 150 140 130 120 110 100 90 80 70 60 50 40 30 20 ppm

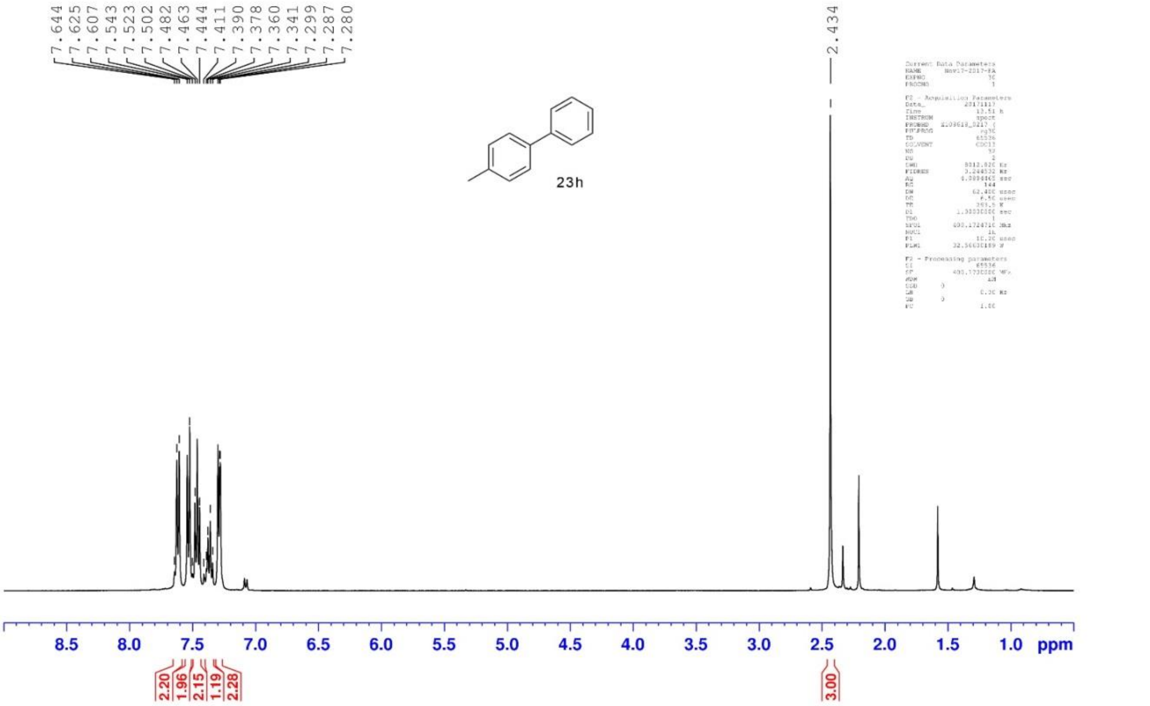
7.644
7.625
7.607
7.543
7.523
7.502
7.482
7.463
7.444
7.411
7.390
7.378
7.360
7.341
7.299
7.287
7.280
2.434
23h
8.5 8.0 7.5 7.0 6.5 6.0 5.5 5.0 4.5 4.0 3.5 3.0 2.5 2.0 1.5 1.0 ppm
2.20
1.96
2.15
1.19
2.28
3.00

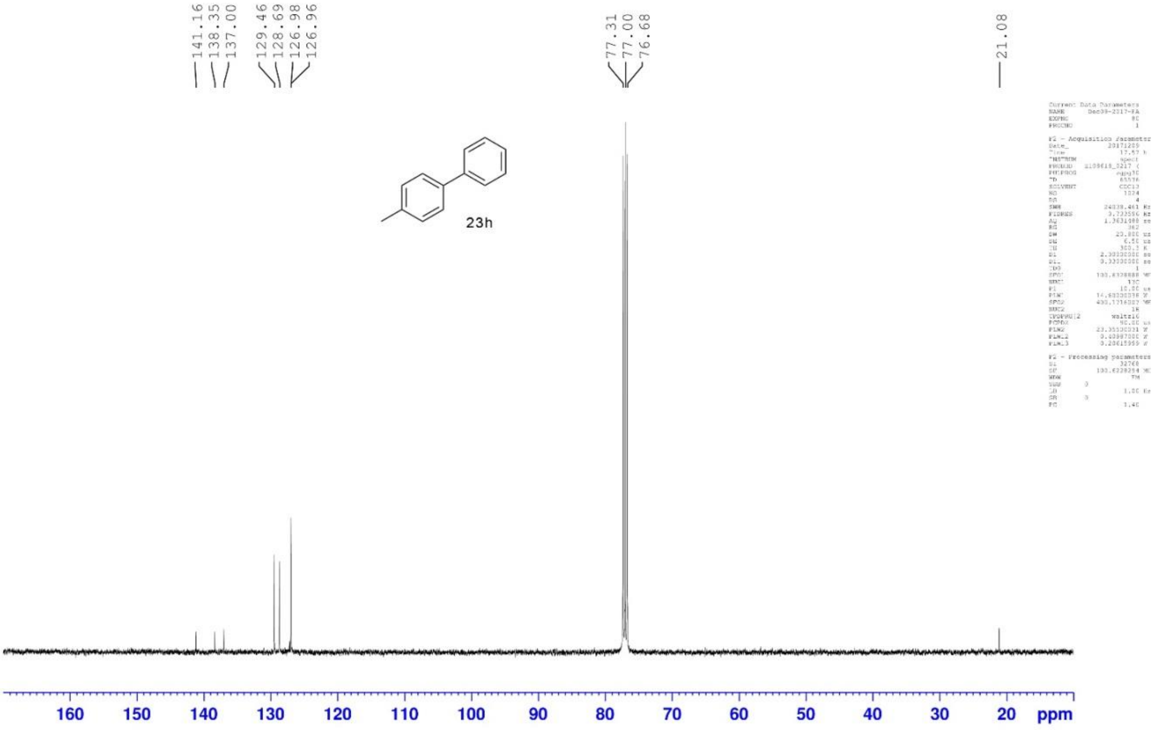
141.16
138.35
137.00
129.46
128.69
126.98
126.96
77.31
77.00
76.68
21.08
23h
160 150 140 130 120 110 100 90 80 70 60 50 40 30 20 ppm

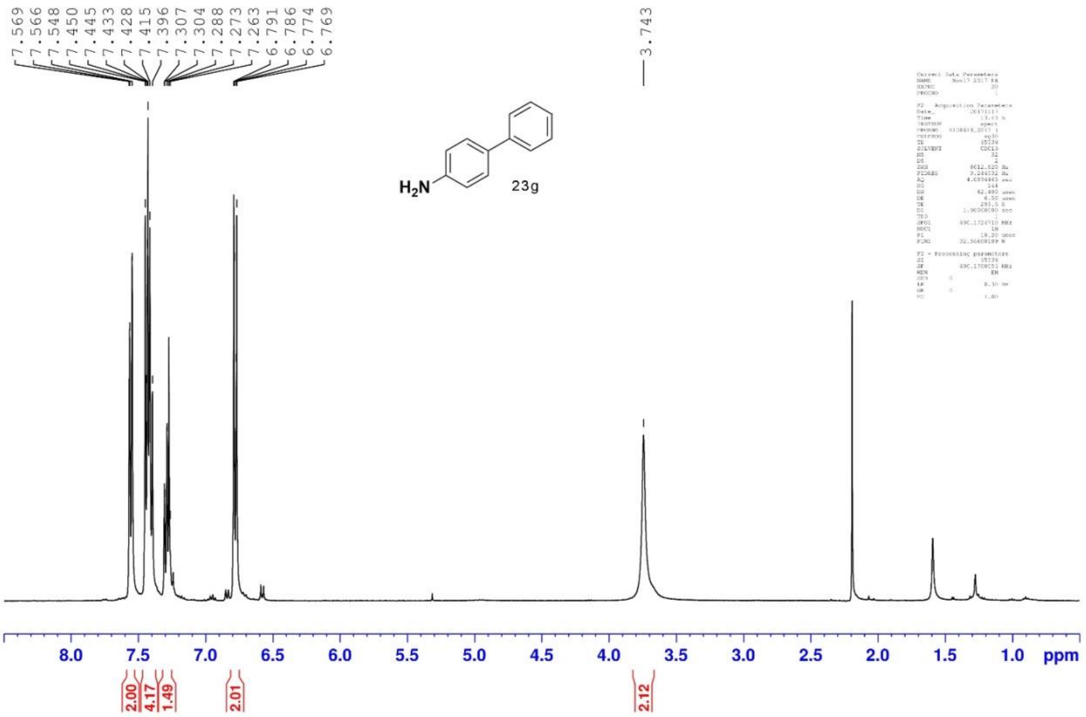
7.569
7.566
7.548
7.450
7.445
7.433
7.428
7.415
7.396
7.307
7.304
7.288
7.273
7.263
6.791
6.786
6.774
6.769
3.743
H_2N
23g
8.0
7.5
7.0
6.5
6.0
5.5
5.0
4.5
4.0
3.5
3.0
2.5
2.0
1.5
1.0
ppm
2.00
4.17
1.49
2.01
2.12

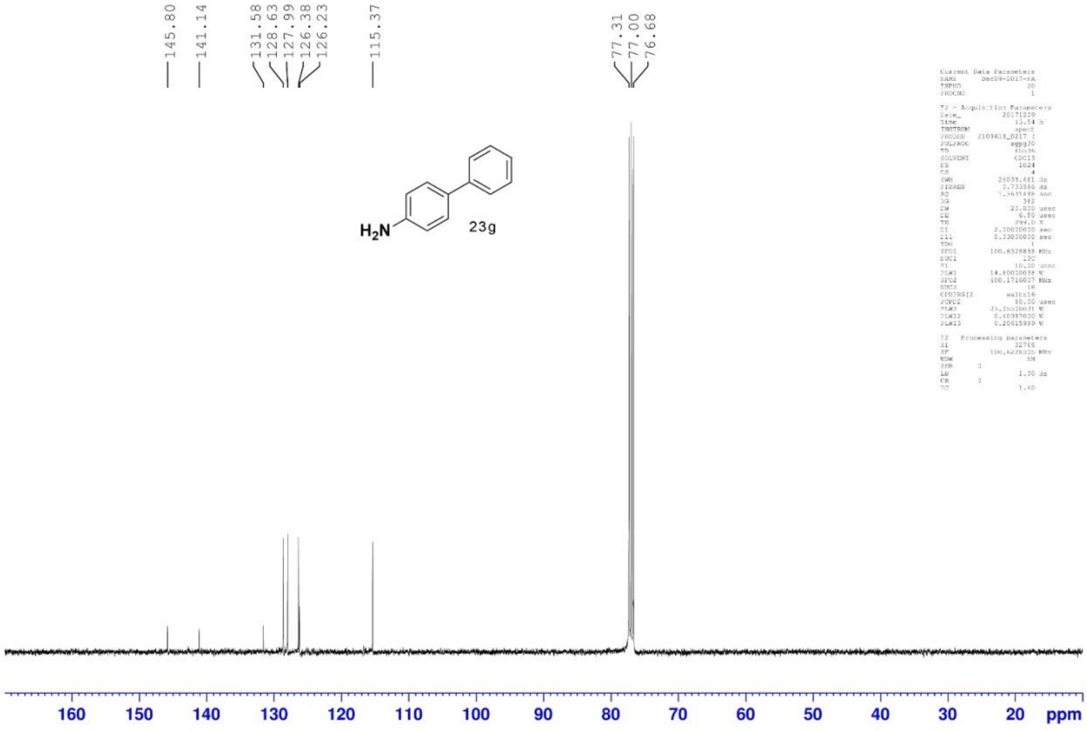
145.80
141.14
131.58
128.63
127.99
126.38
126.23
115.37
77.31
77.00
76.68
H_2N
23g
160
150
140
130
120
110
100
90
80
70
60
50
40
30
20
ppm

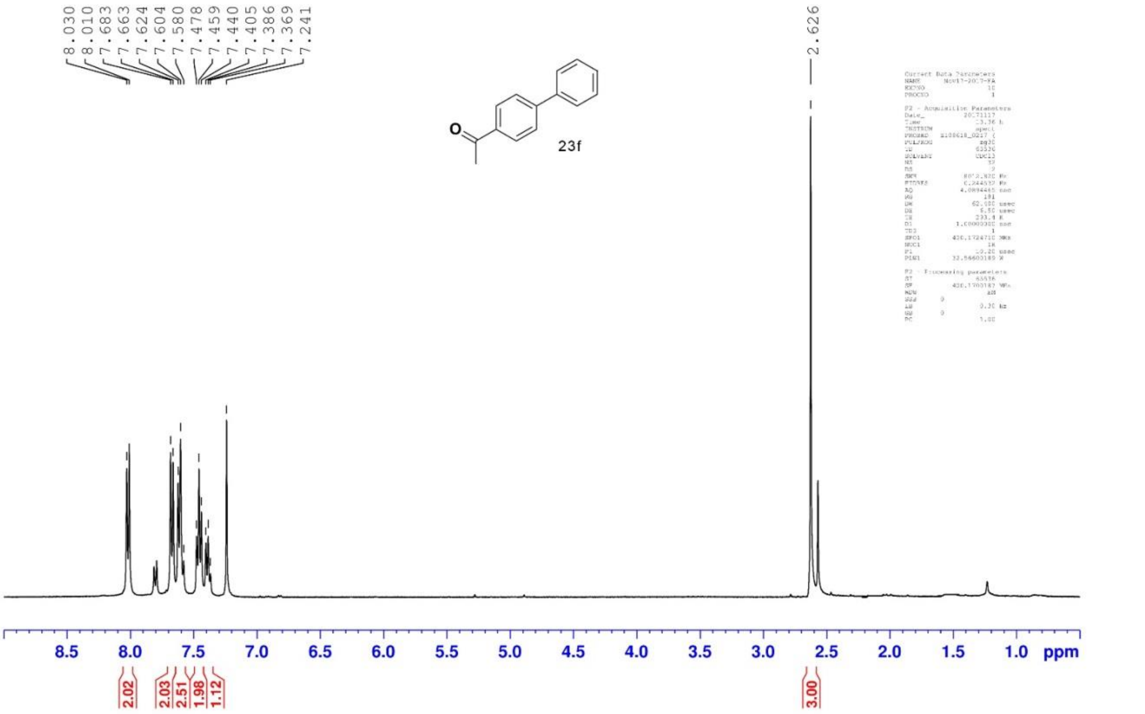
8.030
8.010
7.683
7.663
7.624
7.604
7.580
7.478
7.459
7.440
7.405
7.386
7.369
7.241
2.626
23f
8.5 8.0 7.5 7.0 6.5 6.0 5.5 5.0 4.5 4.0 3.5 3.0 2.5 2.0 1.5 1.0 ppm
2.02
2.03
2.51
1.98
1.12
3.00

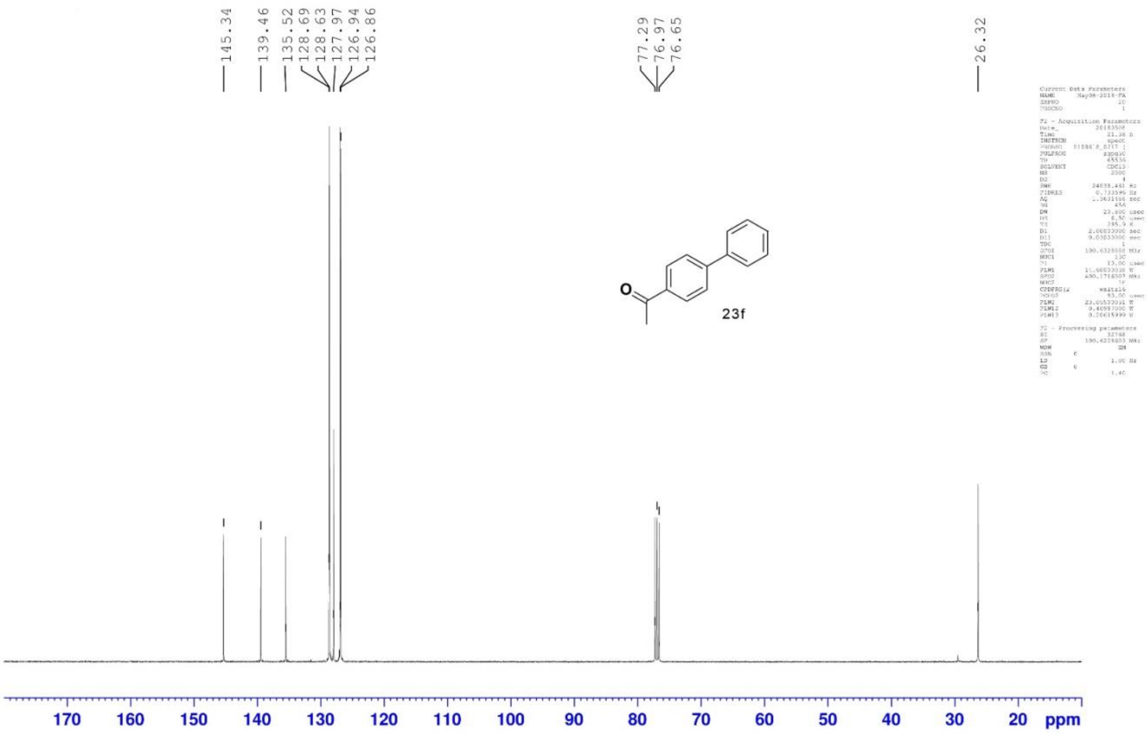
145.34
139.46
135.52
128.69
128.63
127.97
126.94
126.86
77.29
76.97
76.65
26.32
23f
170 160 150 140 130 120 110 100 90 80 70 60 50 40 30 20 ppm

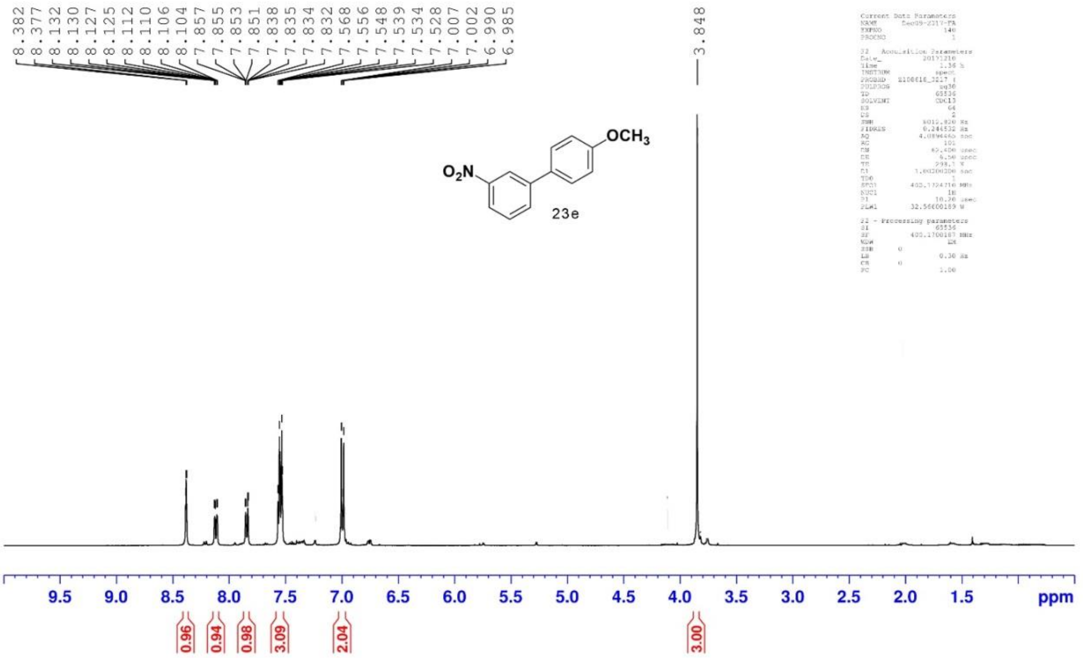
8.382
8.377
8.132
8.130
8.127
8.125
8.112
8.110
8.106
8.104
7.857
7.855
7.853
7.851
7.838
7.835
7.834
7.832
7.568
7.556
7.548
7.539
7.534
7.528
7.007
7.002
6.990
6.985
3.848
OCH3
O2N
23e
9.5
9.0
8.5
8.0
7.5
7.0
6.5
6.0
5.5
5.0
4.5
4.0
3.5
3.0
2.5
2.0
1.5
ppm
0.96
0.94
0.98
3.09
2.04
3.00

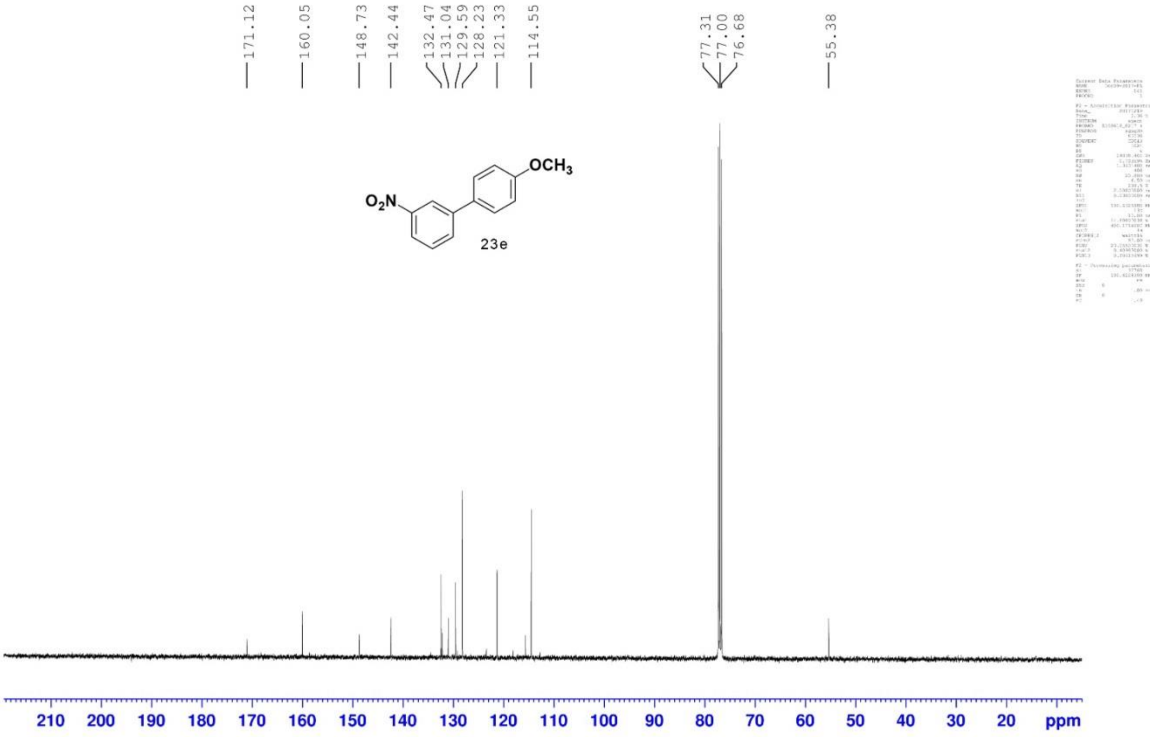
171.12
160.05
148.73
142.44
132.47
131.04
129.59
128.23
121.33
114.55
77.31
77.00
76.68
55.38
OCH3
O2N
23e
210
200
190
180
170
160
150
140
130
120
110
100
90
80
70
60
50
40
30
20
ppm

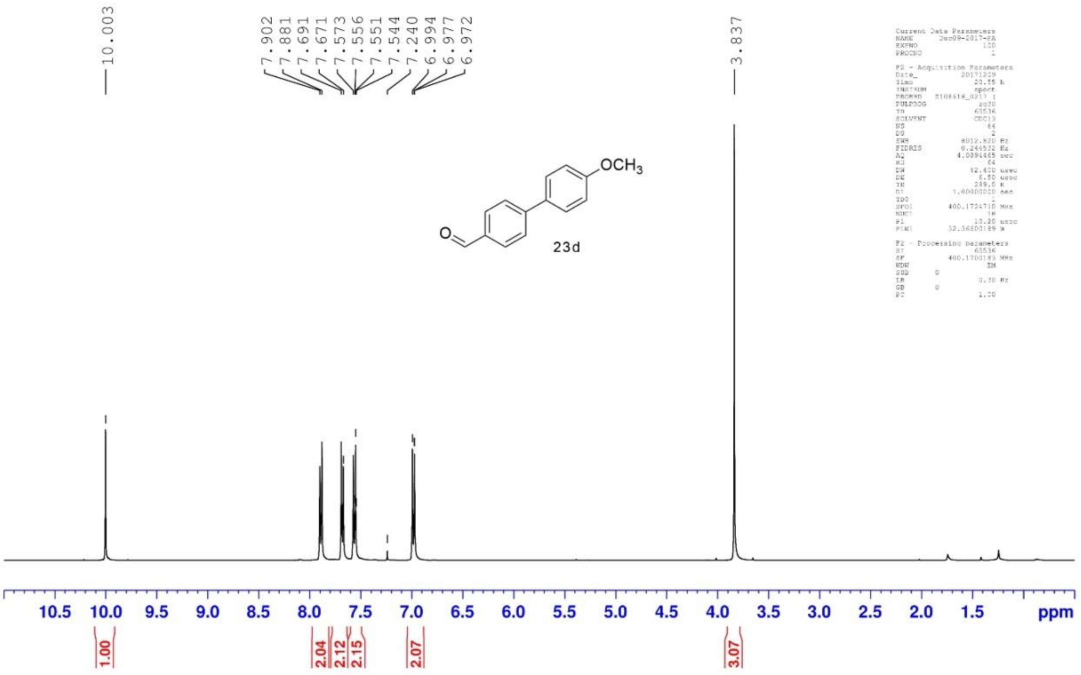
10.003
7.902
7.881
7.691
7.671
7.573
7.556
7.551
7.544
7.240
6.994
6.977
6.972
3.837
OCH3
O
23d
10.5 10.0 9.5 9.0 8.5 8.0 7.5 7.0 6.5 6.0 5.5 5.0 4.5 4.0 3.5 3.0 2.5 2.0 1.5 ppm
1.00
2.04
2.12
2.15
2.07
3.07

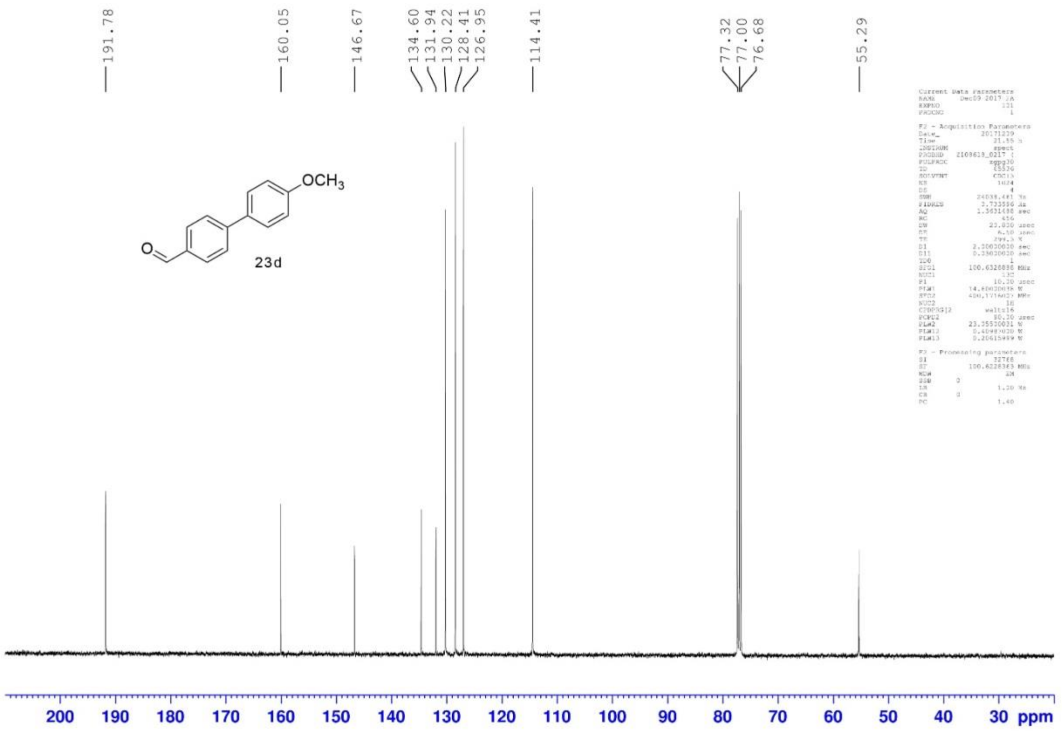
191.78
160.05
146.67
134.60
131.94
130.22
128.41
126.95
114.41
77.32
77.00
76.68
55.29
OCH3
O
23d
200 190 180 170 160 150 140 130 120 110 100 90 80 70 60 50 40 30 ppm

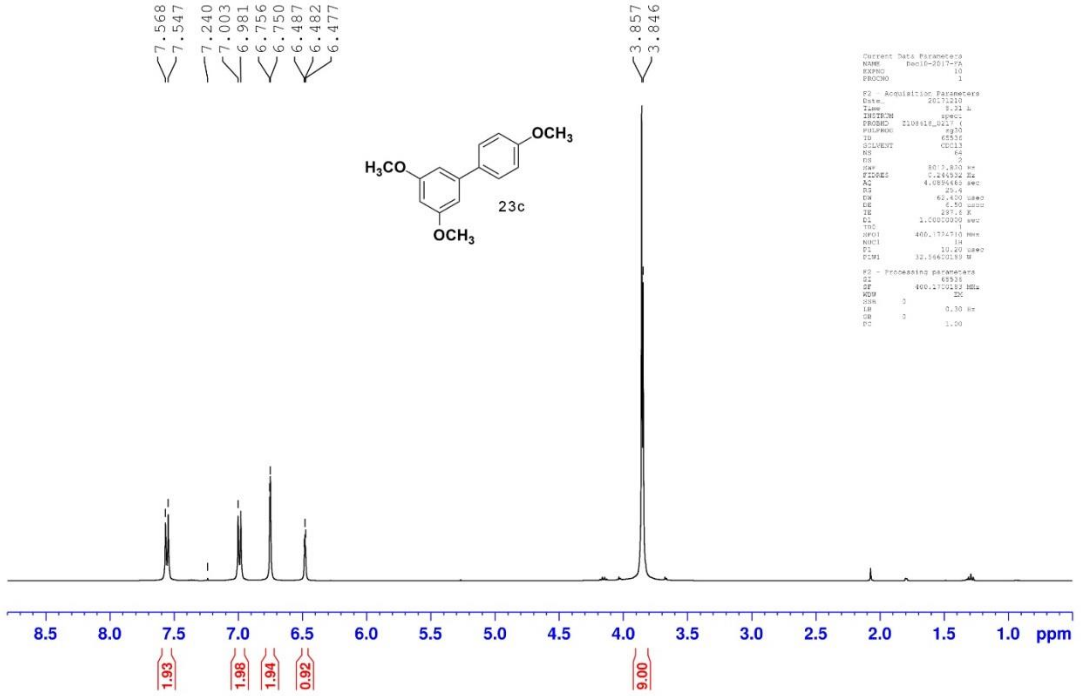
7.568
7.547
7.240
7.003
6.981
6.756
6.750
6.487
6.482
6.477
3.857
3.846
H3CO
OCH3
OCH3
23c
8.5 8.0 7.5 7.0 6.5 6.0 5.5 5.0 4.5 4.0 3.5 3.0 2.5 2.0 1.5 1.0 ppm
1.93
1.98
1.94
0.92
9.00

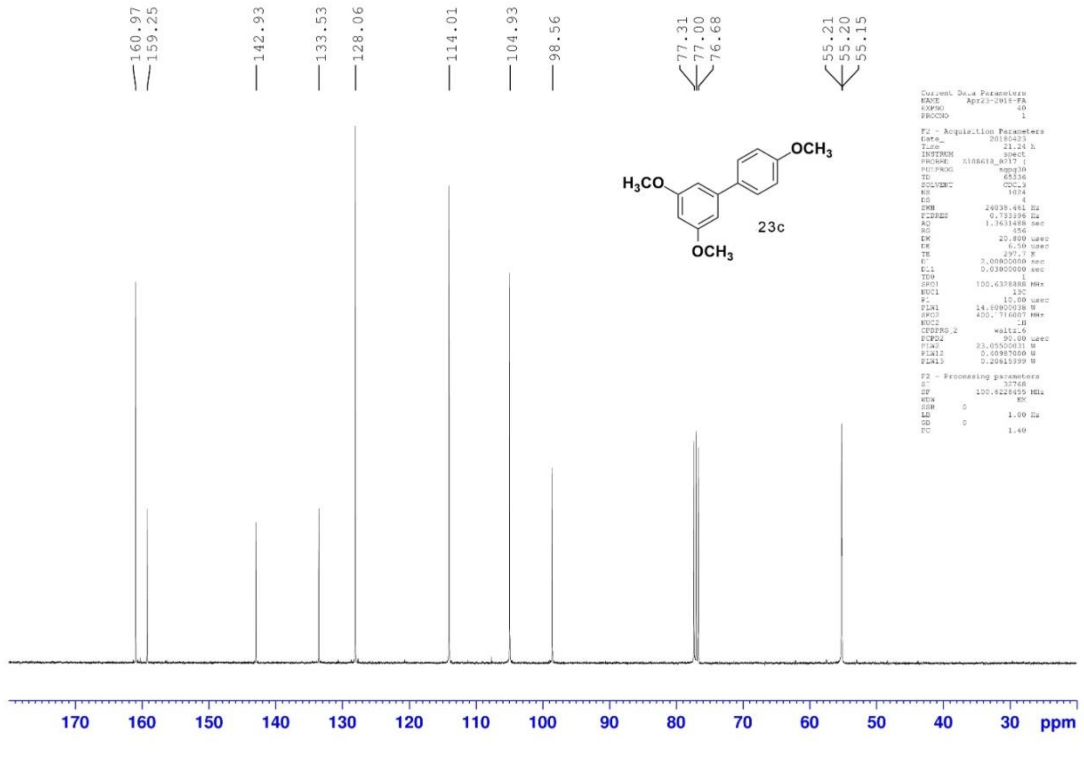
160.97
159.25
142.93
133.53
128.06
114.01
104.93
98.56
77.31
77.00
76.68
55.21
55.20
55.15
H3CO
OCH3
OCH3
23c
170 160 150 140 130 120 110 100 90 80 70 60 50 40 30 ppm

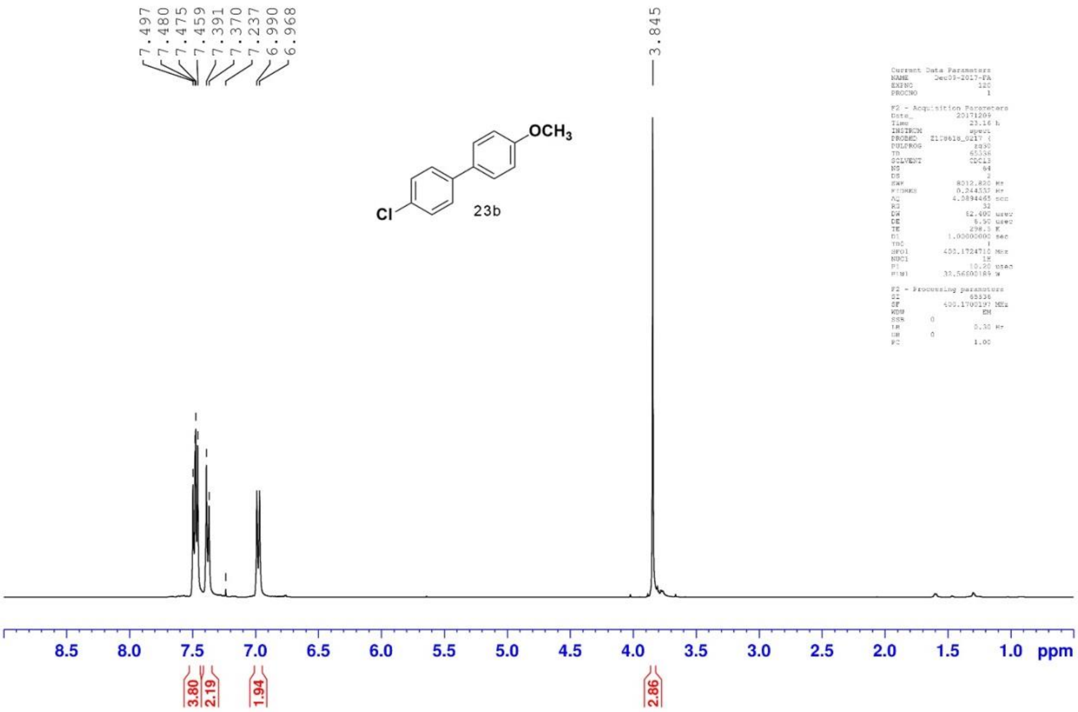

7.497
7.480
7.475
7.459
7.391
7.370
7.237
6.990
6.968
3.845
OCH3
Cl
23b
8.5 8.0 7.5 7.0 6.5 6.0 5.5 5.0 4.5 4.0 3.5 3.0 2.5 2.0 1.5 1.0 ppm
3.80
2.19
1.94
2.86

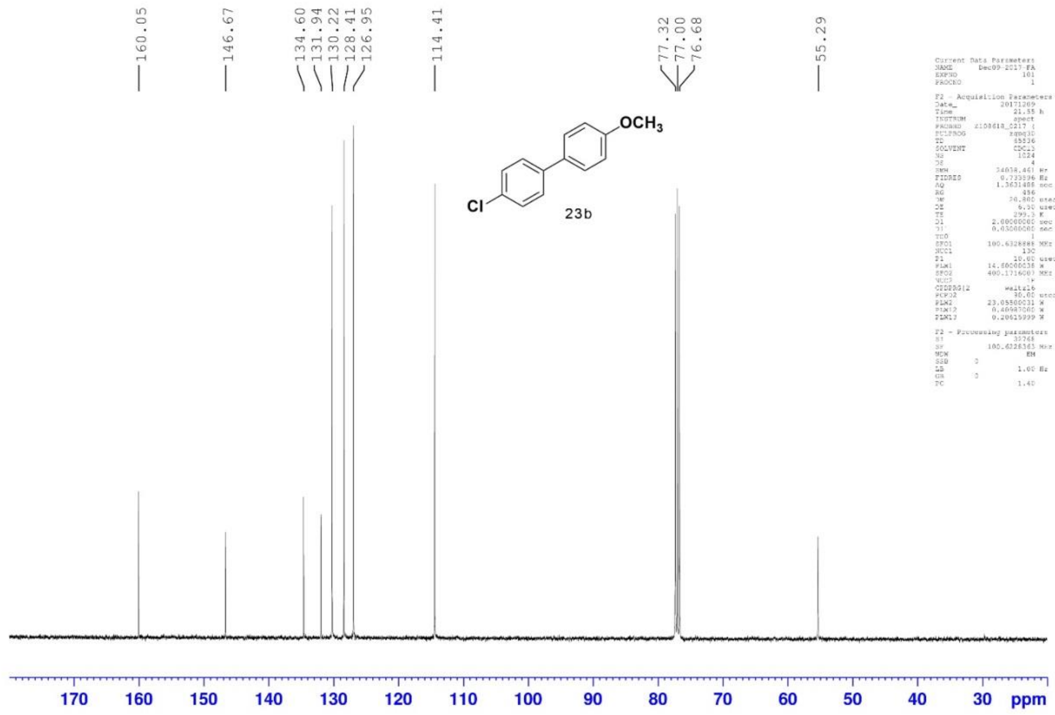

160.05
146.67
134.60
131.94
130.22
128.41
126.95
114.41
77.32
77.00
76.68
55.29
OCH3
Cl
23b
170 160 150 140 130 120 110 100 90 80 70 60 50 40 30 ppm

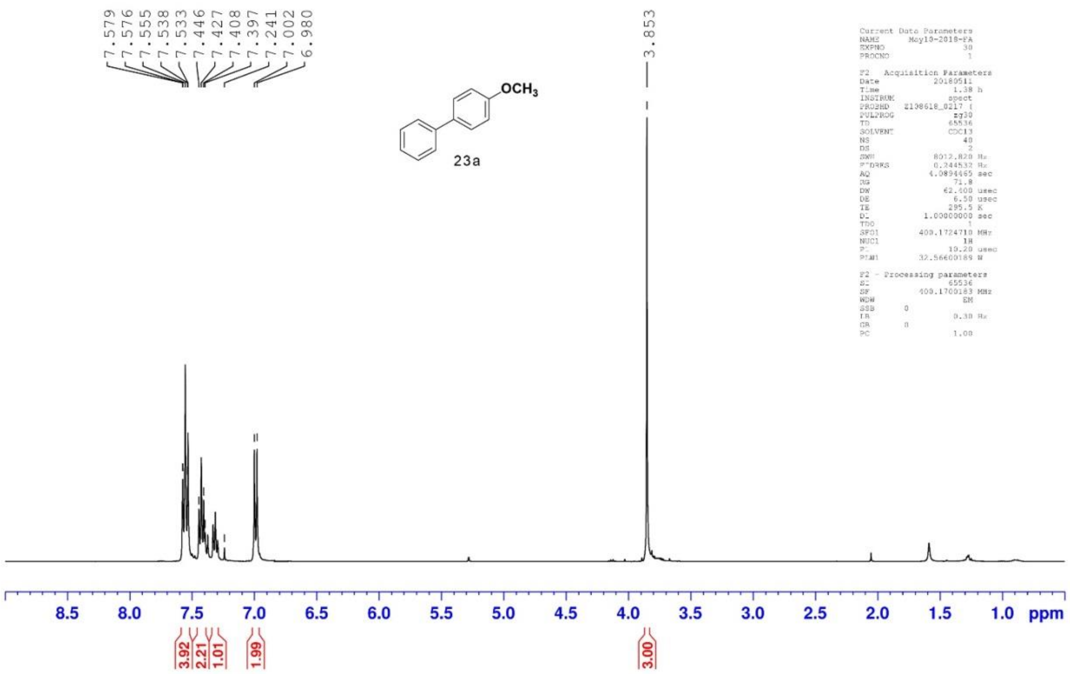
7.579
7.576
7.555
7.538
7.533
7.446
7.427
7.408
7.397
7.241
7.002
6.980
3.853
OCH3
23a
8.5 8.0 7.5 7.0 6.5 6.0 5.5 5.0 4.5 4.0 3.5 3.0 2.5 2.0 1.5 1.0 ppm
3.92
2.21
1.01
1.99
3.00

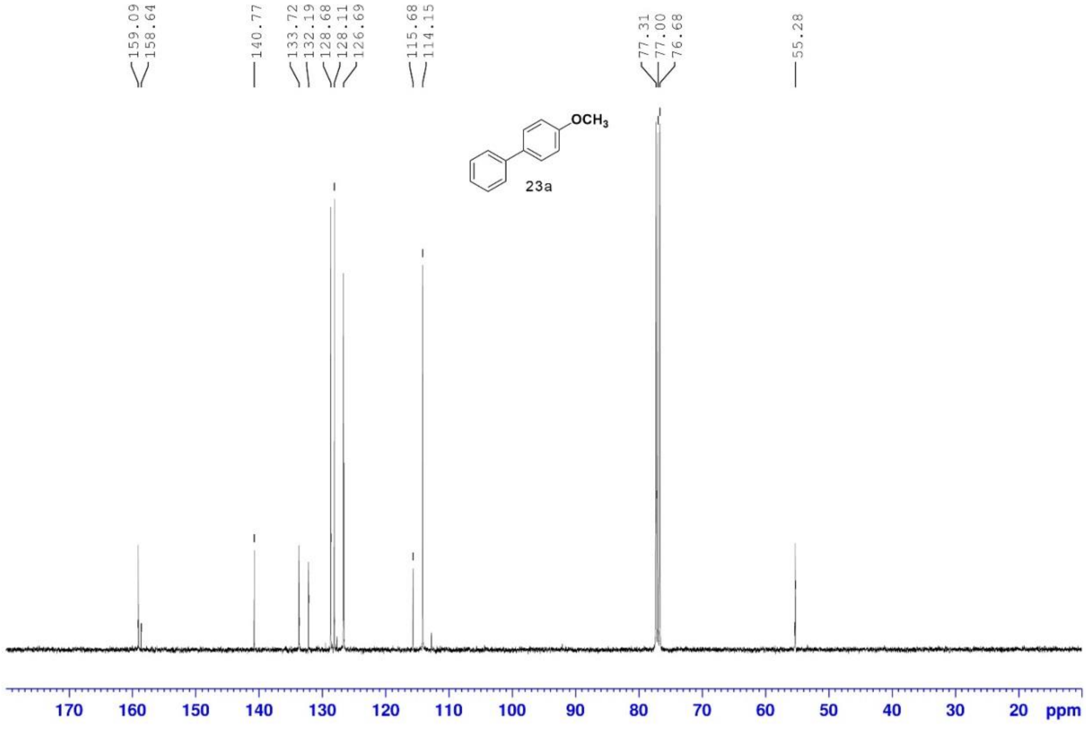
159.09
158.64
140.77
133.72
132.19
128.68
128.11
126.69
115.68
114.15
77.31
77.00
76.68
55.28
OCH3
23a
170 160 150 140 130 120 110 100 90 80 70 60 50 40 30 20 ppm

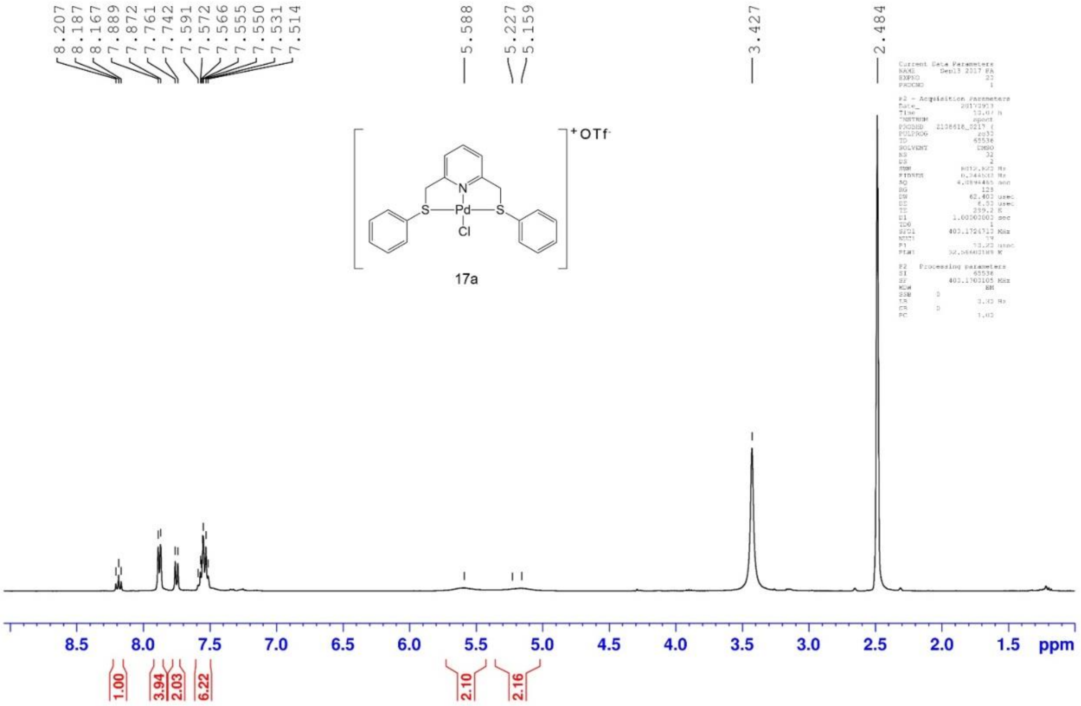
8.207
8.187
8.167
7.889
7.872
7.761
7.742
7.591
7.572
7.566
7.555
7.550
7.531
7.514
5.588
5.227
5.159
3.427
2.484
+OTf-
N
S
Pd
Cl
S
17a
8.5 8.0 7.5 7.0 6.5 6.0 5.5 5.0 4.5 4.0 3.5 3.0 2.5 2.0 1.5 ppm
1.00
3.94
2.03
6.22
2.10
2.16

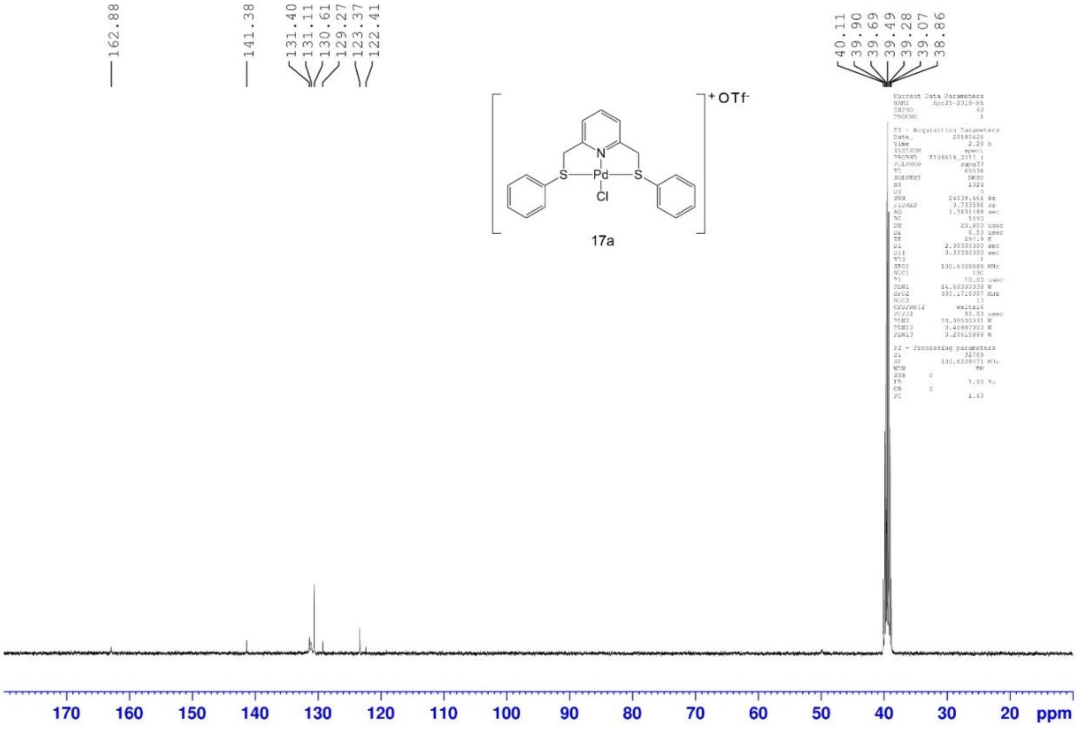
162.88
141.38
131.40
131.11
130.61
129.27
123.37
122.41
40.11
39.90
39.69
39.49
39.28
39.07
38.86
+OTf-
N
S
Pd
Cl
S
17a
170 160 150 140 130 120 110 100 90 80 70 60 50 40 30 20 ppm

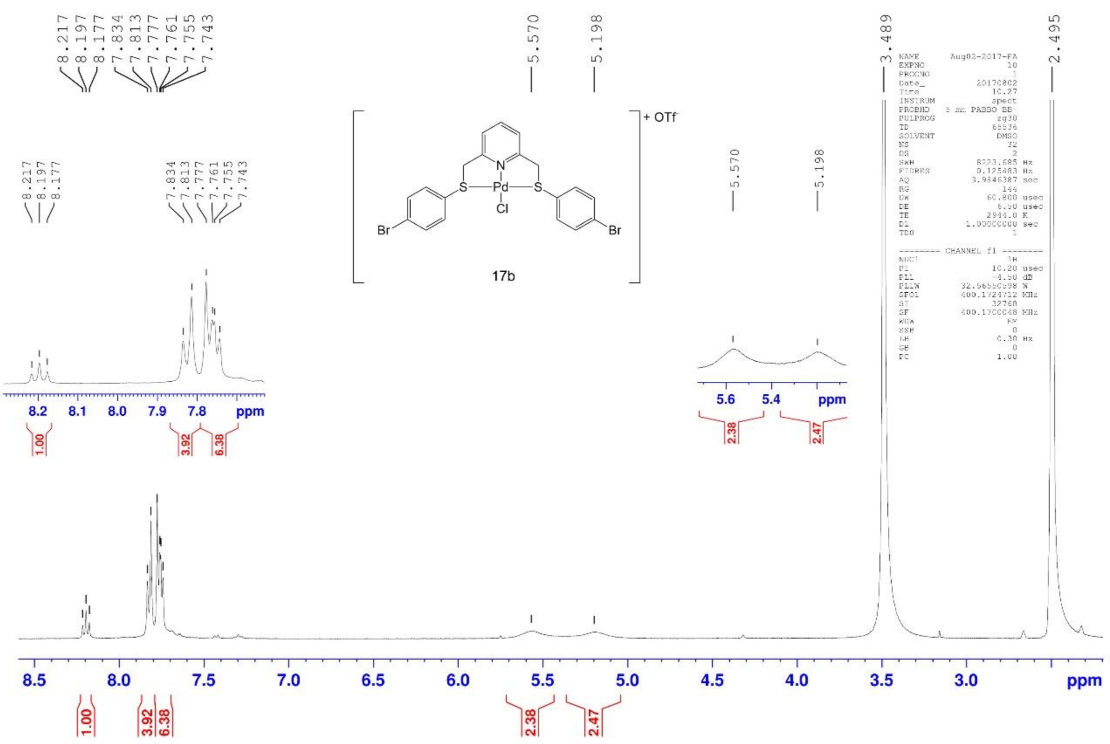

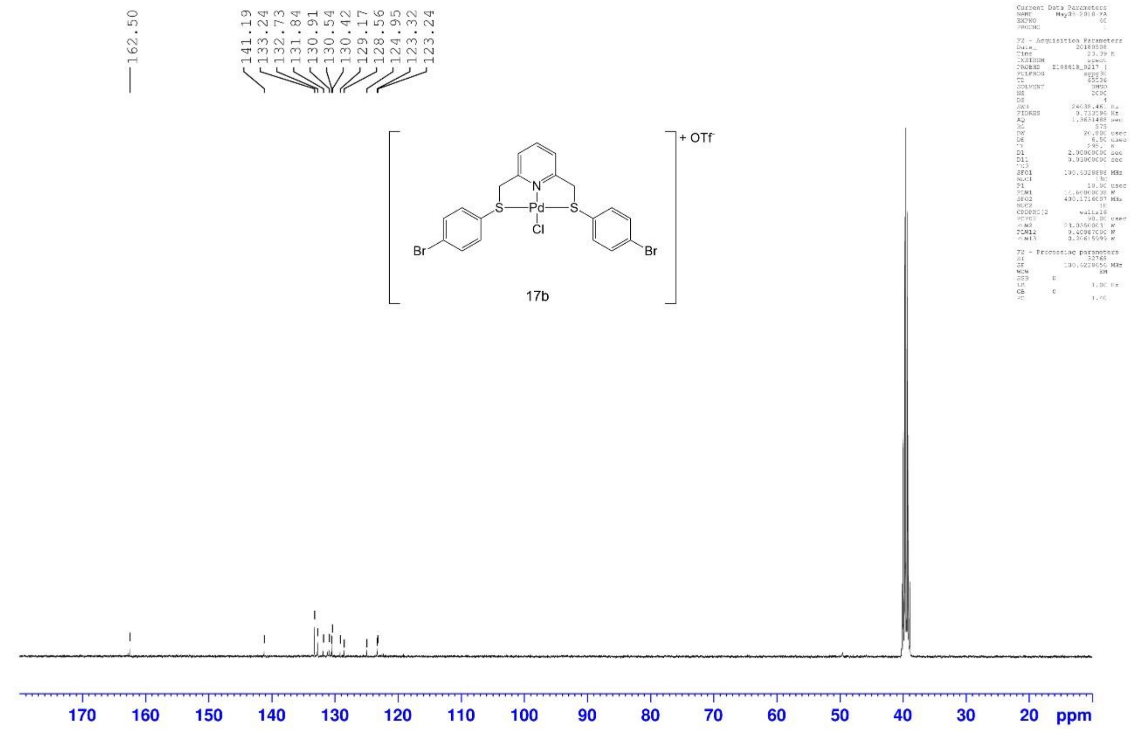

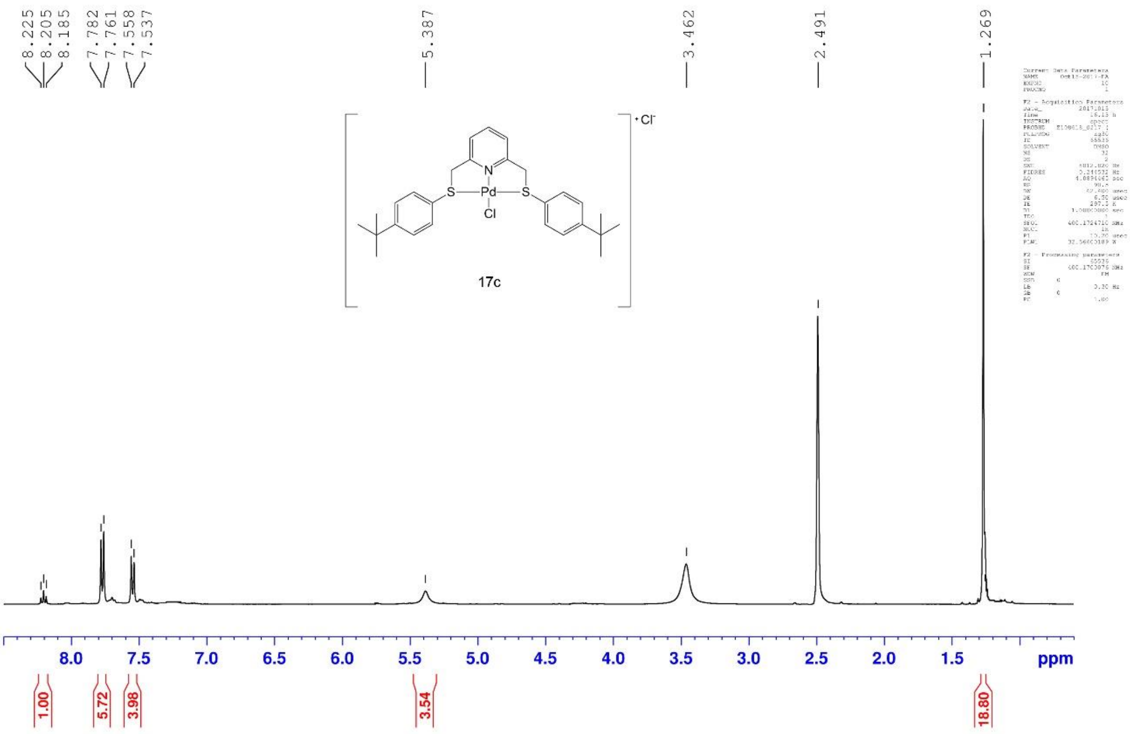

8.225
8.205
8.185
7.782
7.761
7.558
7.537
5.387
3.462
2.491
1.269
Cl⁻
S
Pd
N
Cl
S
17c
8.0
7.5
7.0
6.5
6.0
5.5
5.0
4.5
4.0
3.5
3.0
2.5
2.0
1.5
ppm
1.00
5.72
3.98
3.54
18.80

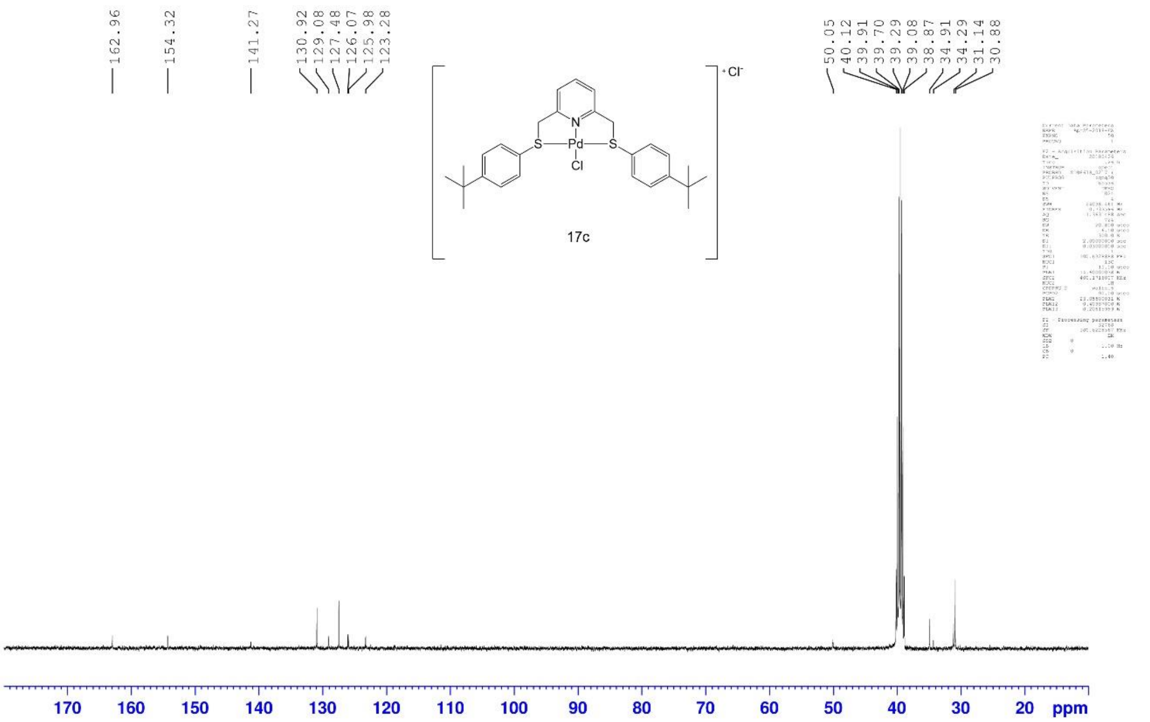

162.96
154.32
141.27
130.92
129.08
127.48
126.07
125.98
123.28
50.05
40.12
39.91
39.70
39.29
39.08
38.87
34.91
34.29
31.14
30.88
Cl⁻
S
Pd
N
Cl
S
17c
170
160
150
140
130
120
110
100
90
80
70
60
50
40
30
20
ppm

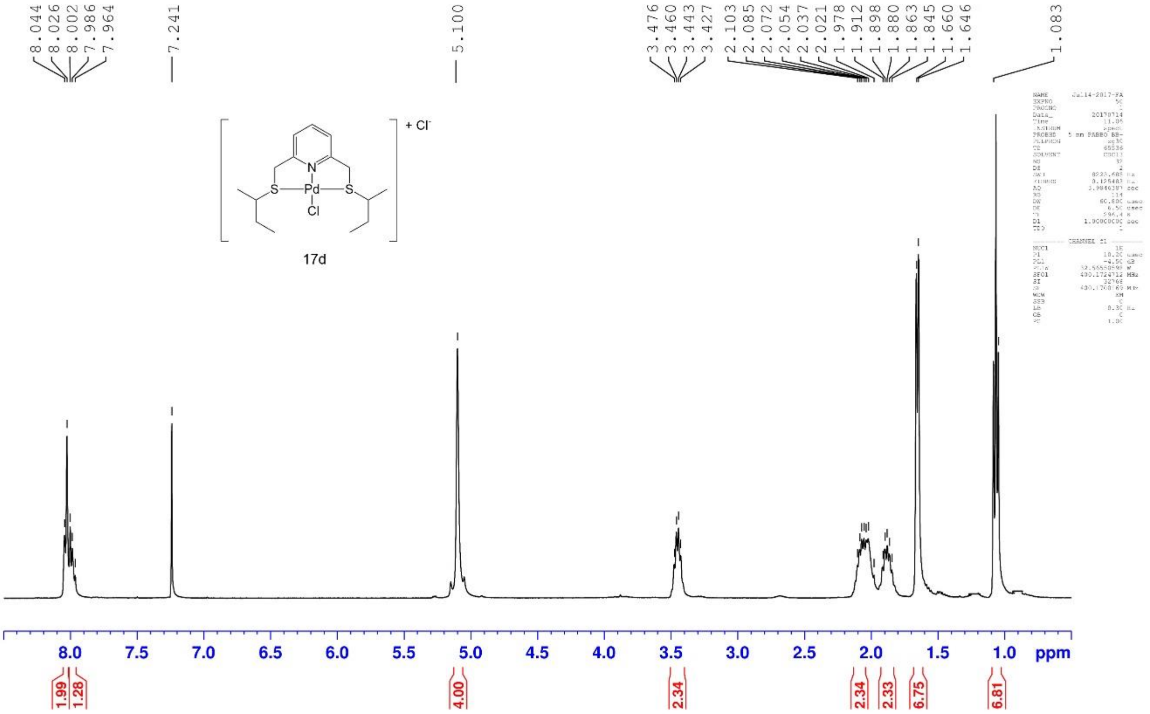
8.044
8.026
8.002
7.986
7.964
7.241
5.100
3.476
3.460
3.443
3.427
2.103
2.085
2.072
2.054
2.037
2.021
1.978
1.912
1.898
1.880
1.863
1.845
1.660
1.646
1.083
+ Cl⁻
N
S
Pd
S
Cl
17d
8.0 7.5 7.0 6.5 6.0 5.5 5.0 4.5 4.0 3.5 3.0 2.5 2.0 1.5 1.0 ppm
1.99
1.28
4.00
2.34
2.34
2.33
6.75
6.81

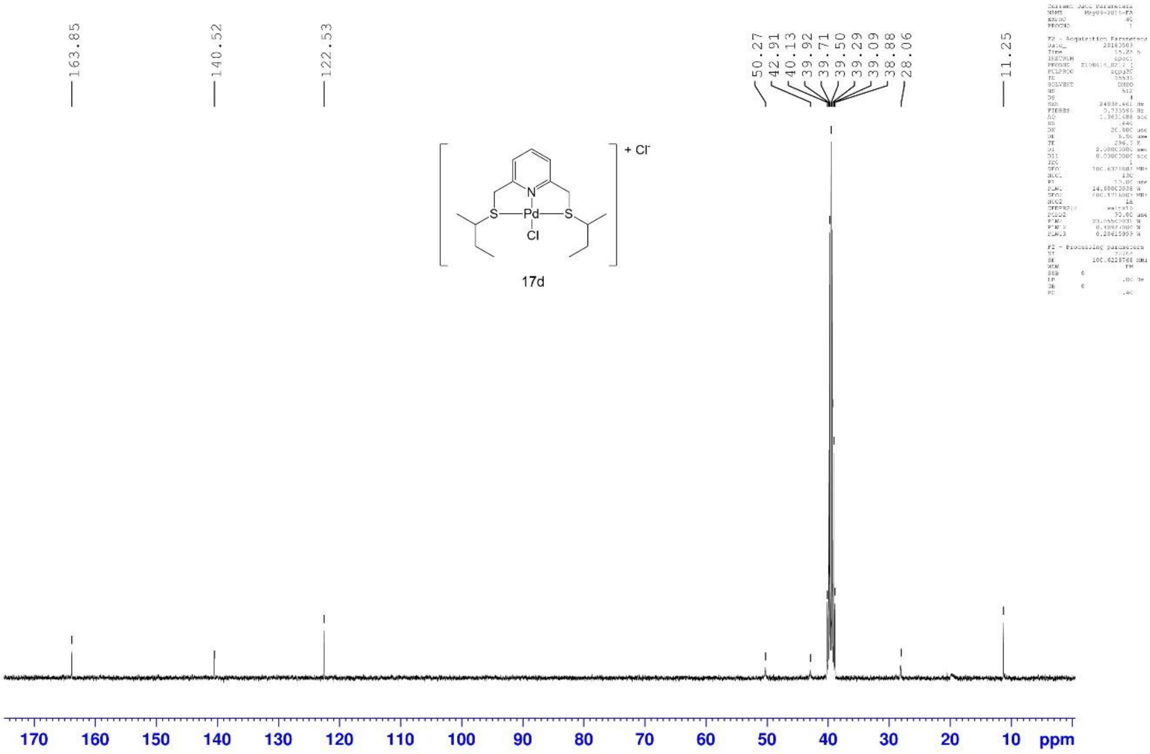
163.85
140.52
122.53
50.27
42.91
40.13
39.92
39.71
39.50
39.29
39.09
38.88
28.06
11.25
+ Cl⁻
N
S
Pd
S
Cl
17d
170 160 150 140 130 120 110 100 90 80 70 60 50 40 30 20 10 ppm

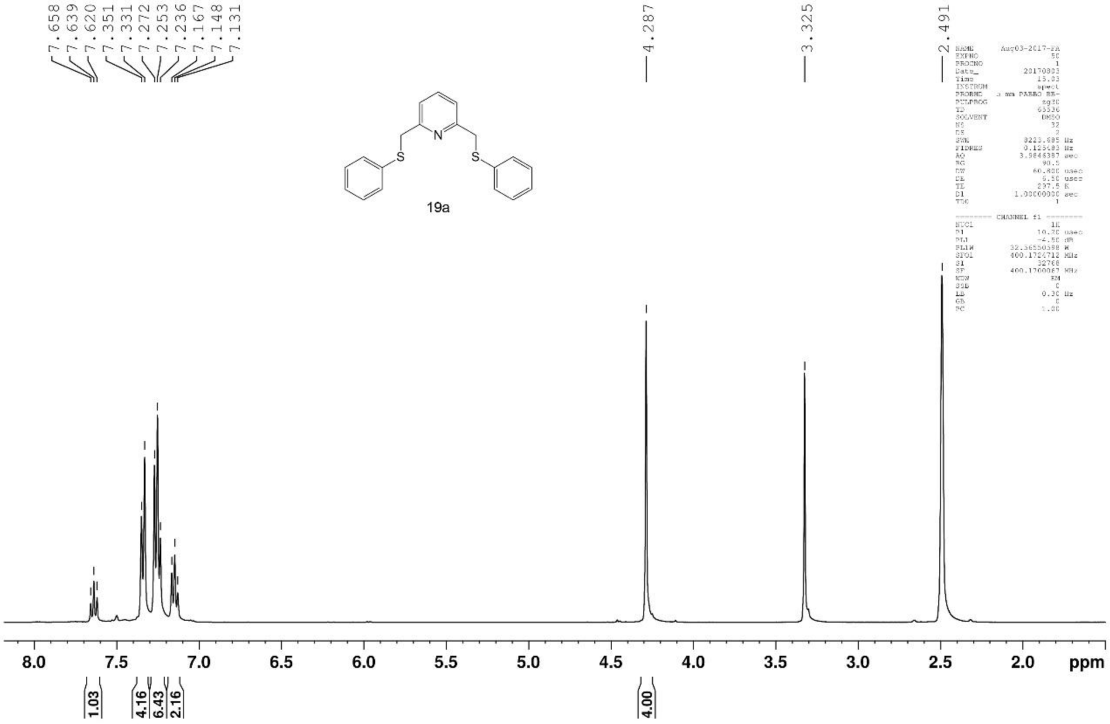
7.658
7.639
7.620
7.351
7.331
7.272
7.253
7.236
7.167
7.148
7.131
4.287
3.325
2.491
19a
8.0 7.5 7.0 6.5 6.0 5.5 5.0 4.5 4.0 3.5 3.0 2.5 2.0 ppm
1.03
4.16
6.43
2.16
4.00

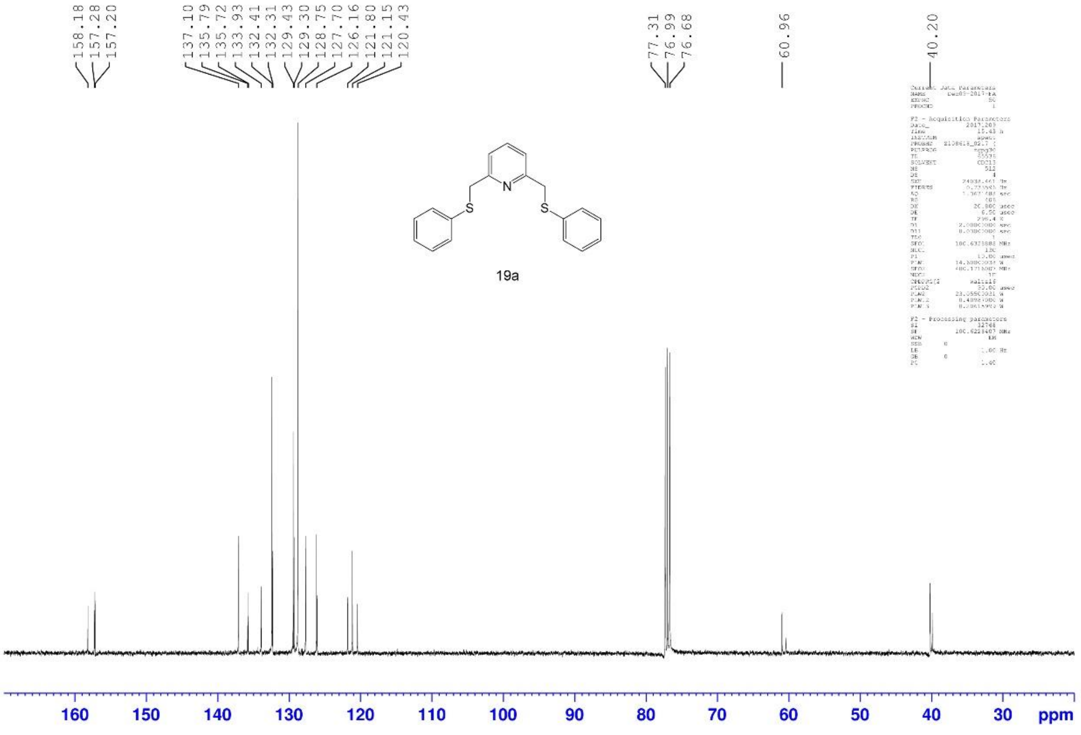
158.18
157.28
157.20
137.10
135.79
135.72
133.93
132.41
132.31
129.43
129.30
128.75
127.70
126.16
121.80
121.15
120.43
77.31
76.99
76.68
60.96
40.20
19a
160 150 140 130 120 110 100 90 80 70 60 50 40 30 ppm

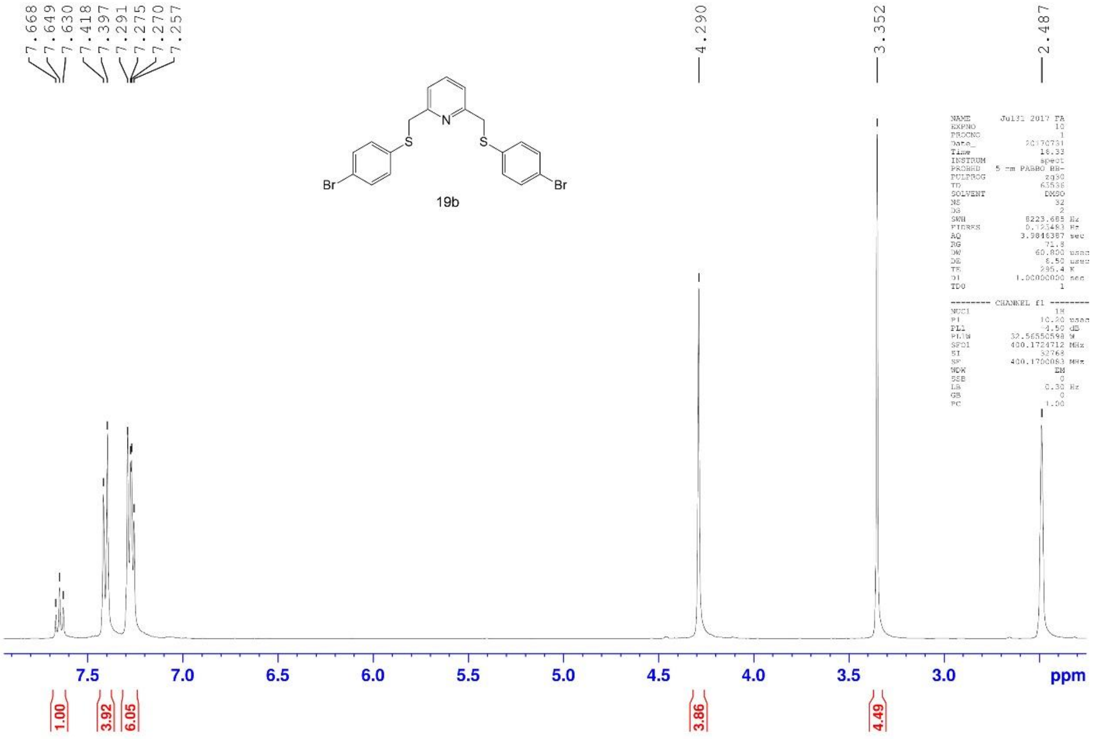
7.668
7.649
7.630
7.418
7.397
7.291
7.275
7.270
7.257
4.290
3.352
2.487
Br
S
N
S
Br
19b
7.5
7.0
6.5
6.0
5.5
5.0
4.5
4.0
3.5
3.0
ppm
1.00
3.92
6.05
3.86
4.49

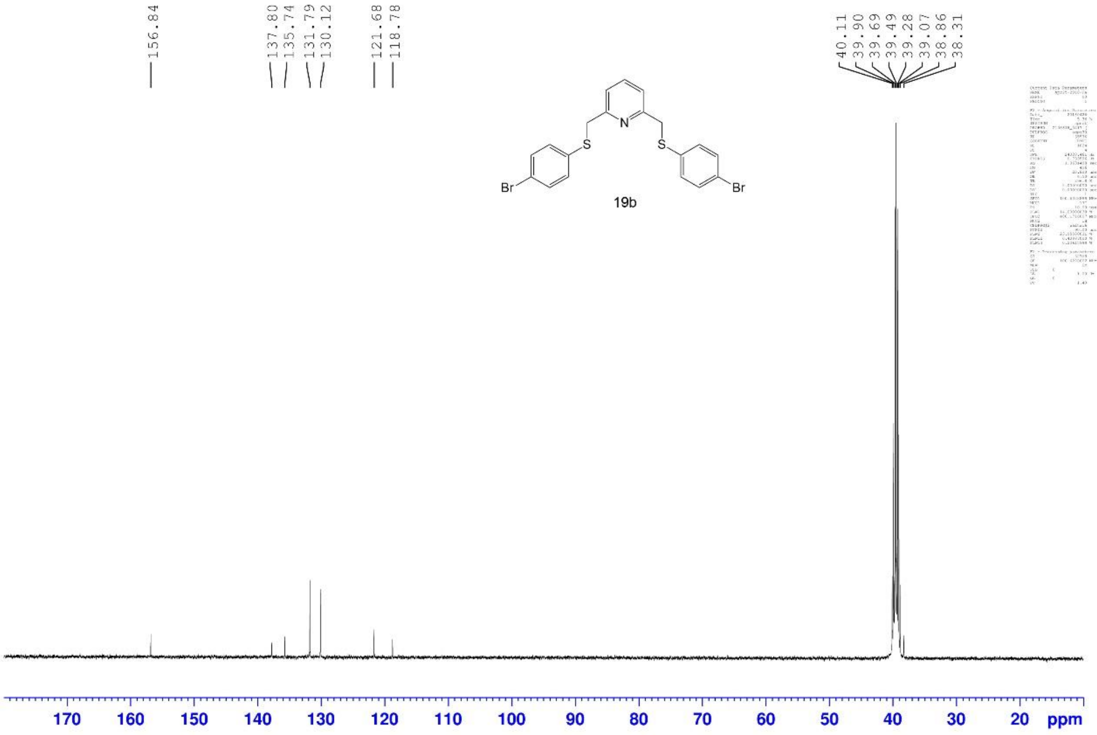
156.84
137.80
135.74
131.79
130.12
121.68
118.78
40.11
39.90
39.69
39.49
39.28
39.07
38.86
38.31
Br
S
N
S
Br
19b
170
160
150
140
130
120
110
100
90
80
70
60
50
40
30
20
ppm

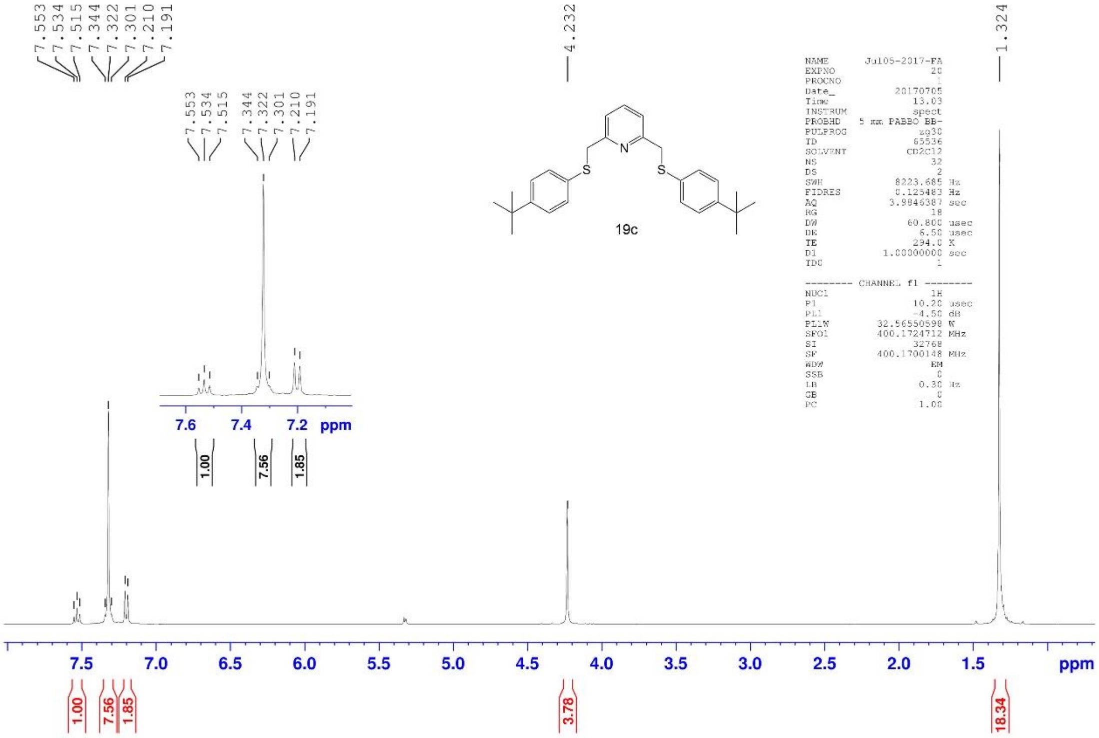
7.553
7.534
7.515
7.344
7.322
7.301
7.210
7.191
4.232
1.324
19c
S
N
7.6 7.4 7.2 ppm
1.00
7.56
1.85
7.5 7.0 6.5 6.0 5.5 5.0 4.5 4.0 3.5 3.0 2.5 2.0 1.5 ppm
3.78
18.34

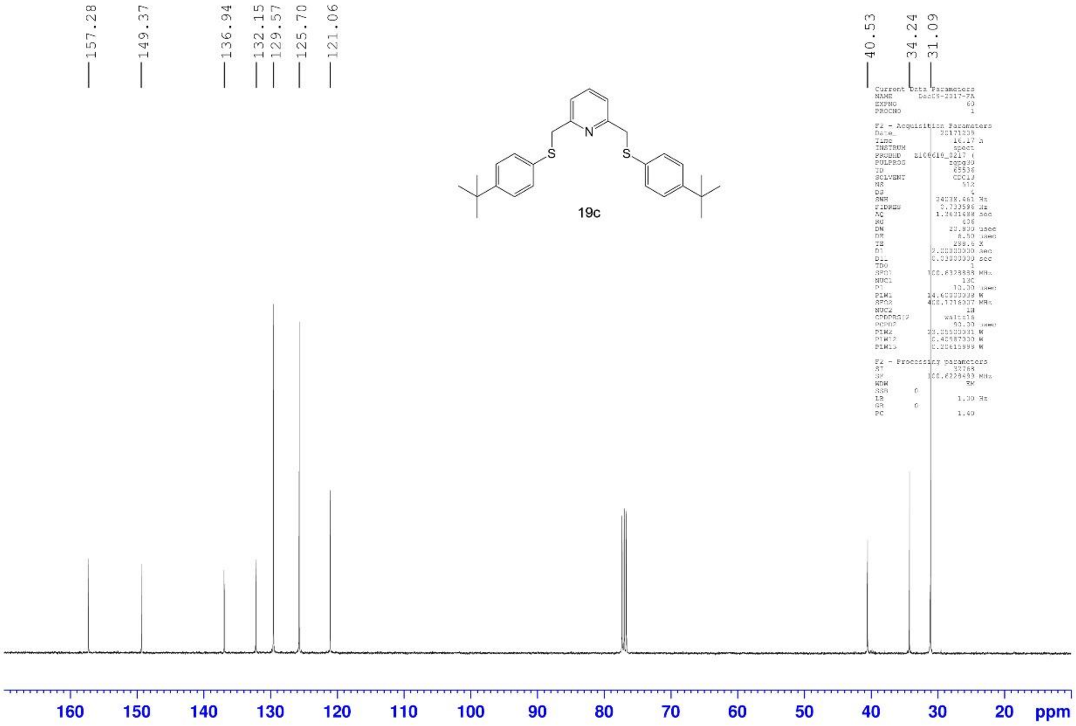
157.28
149.37
136.94
132.15
129.57
125.70
121.06
40.53
34.24
31.09
19c
S
N
160 150 140 130 120 110 100 90 80 70 60 50 40 30 20 ppm

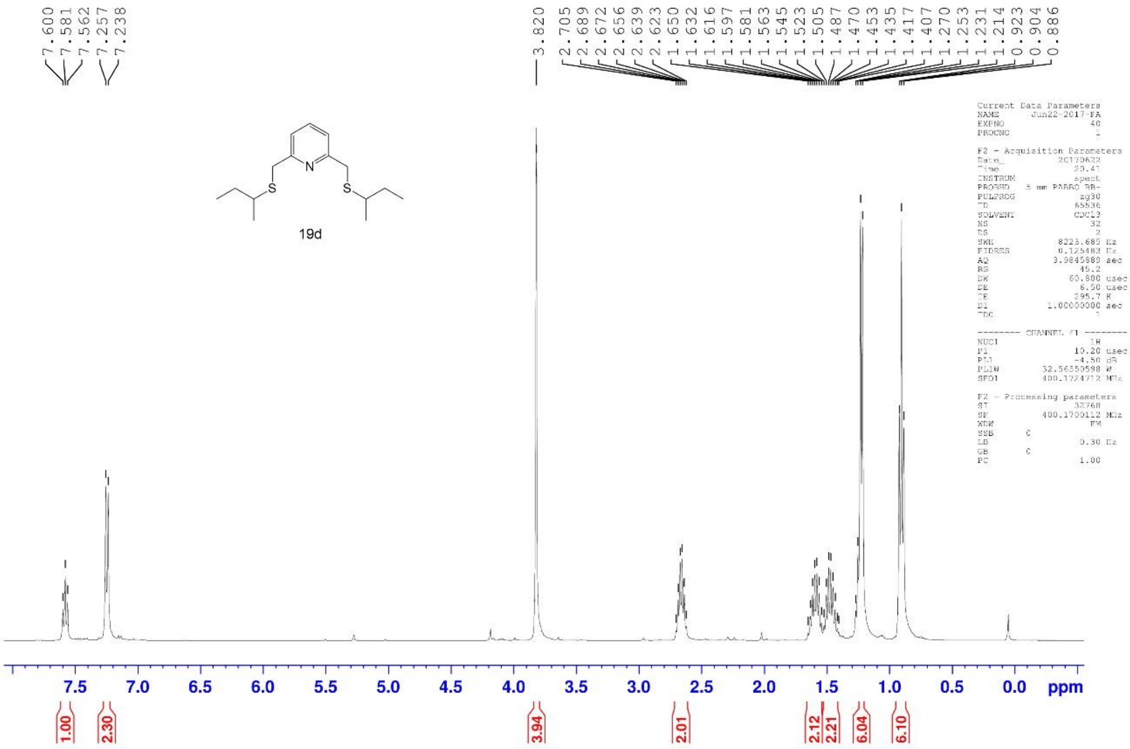

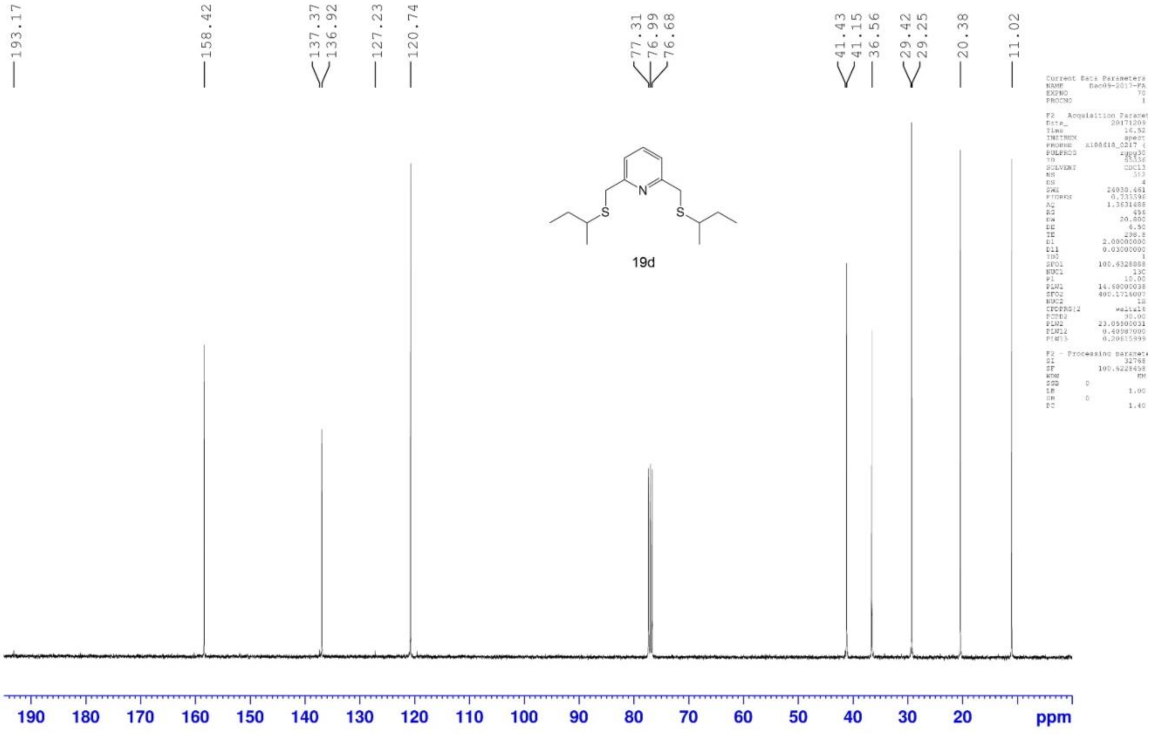

Supplement: File 2 — NMR spectra. [file Beilstein_J_Org_Chem-14-1859-s002.pdf]
